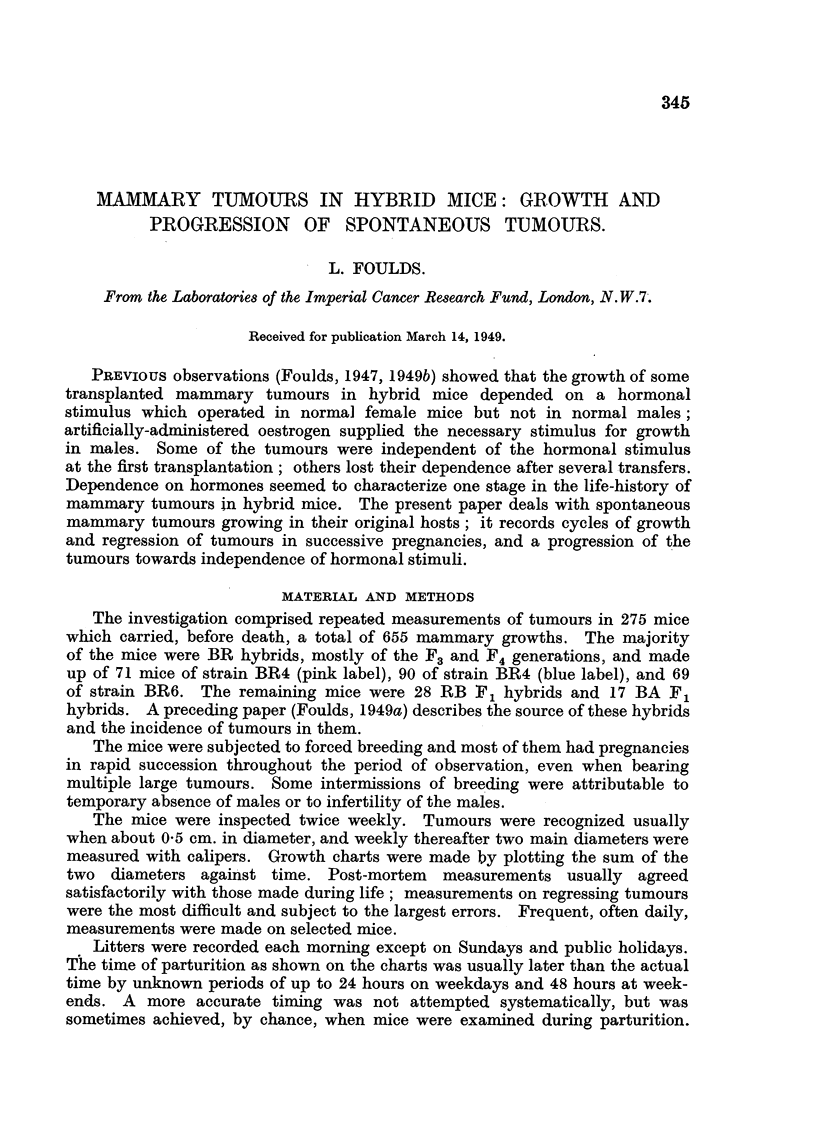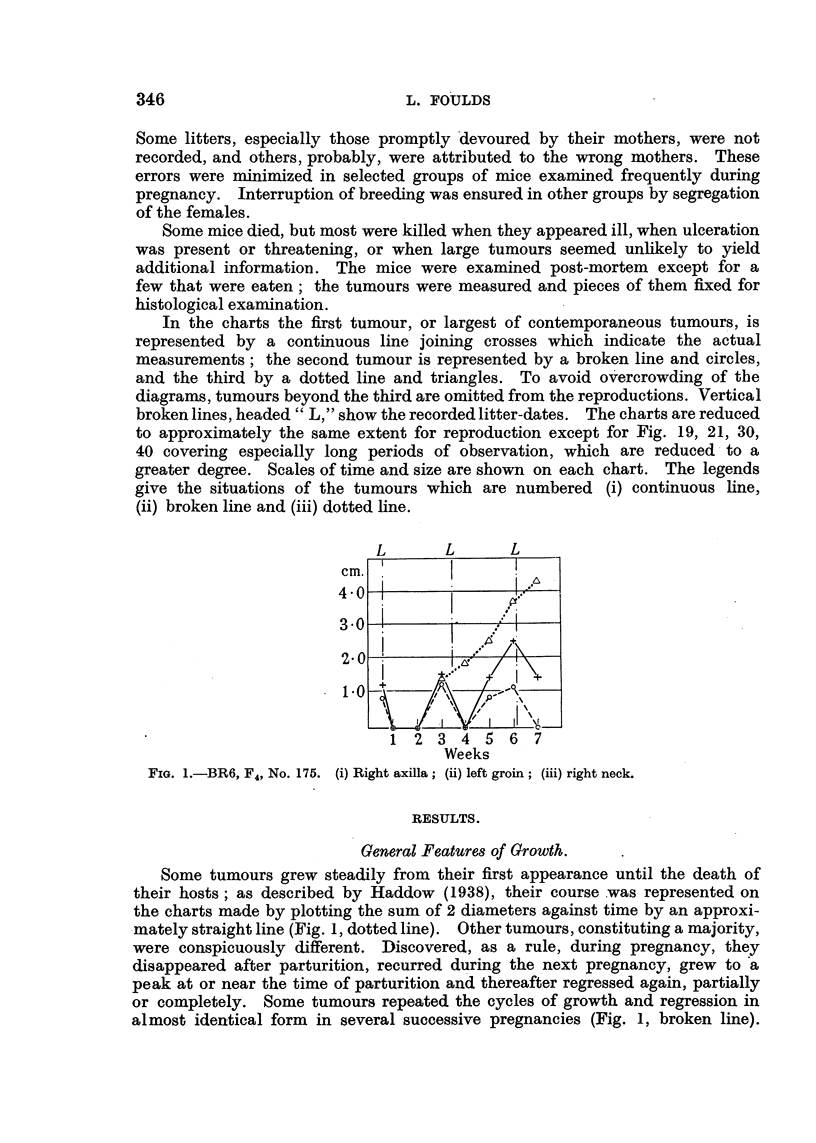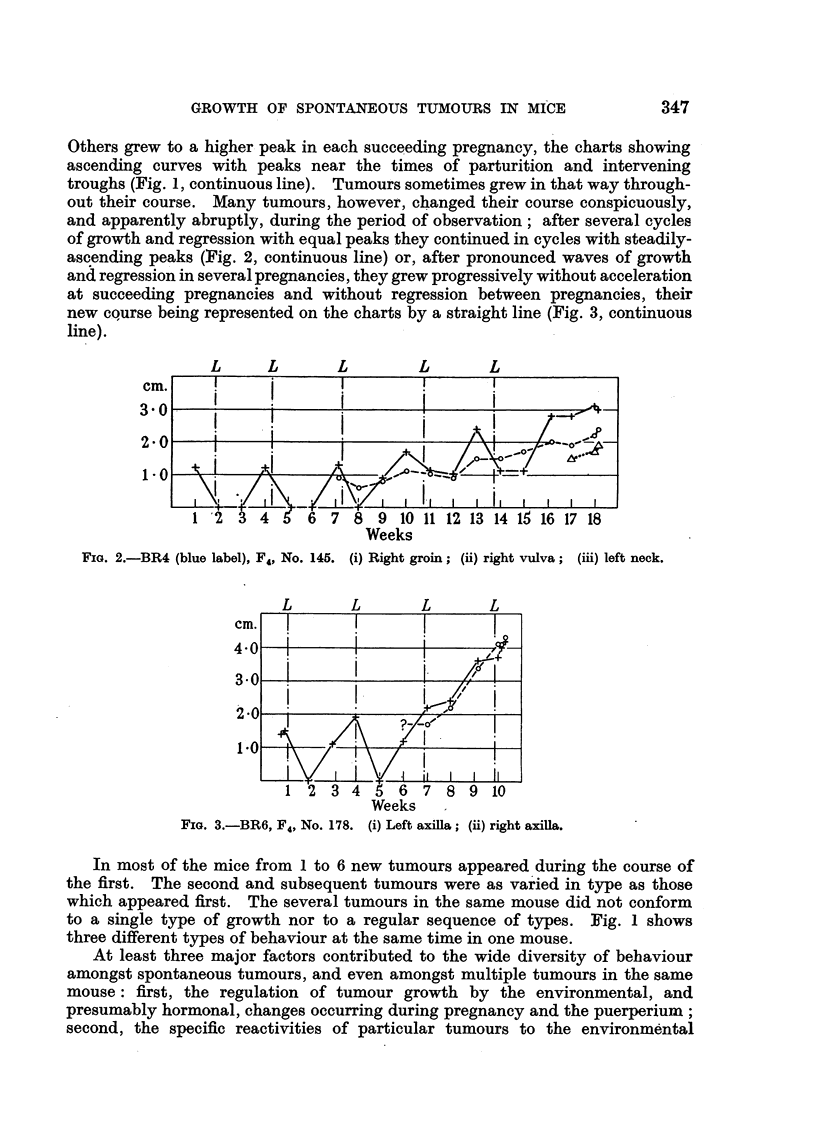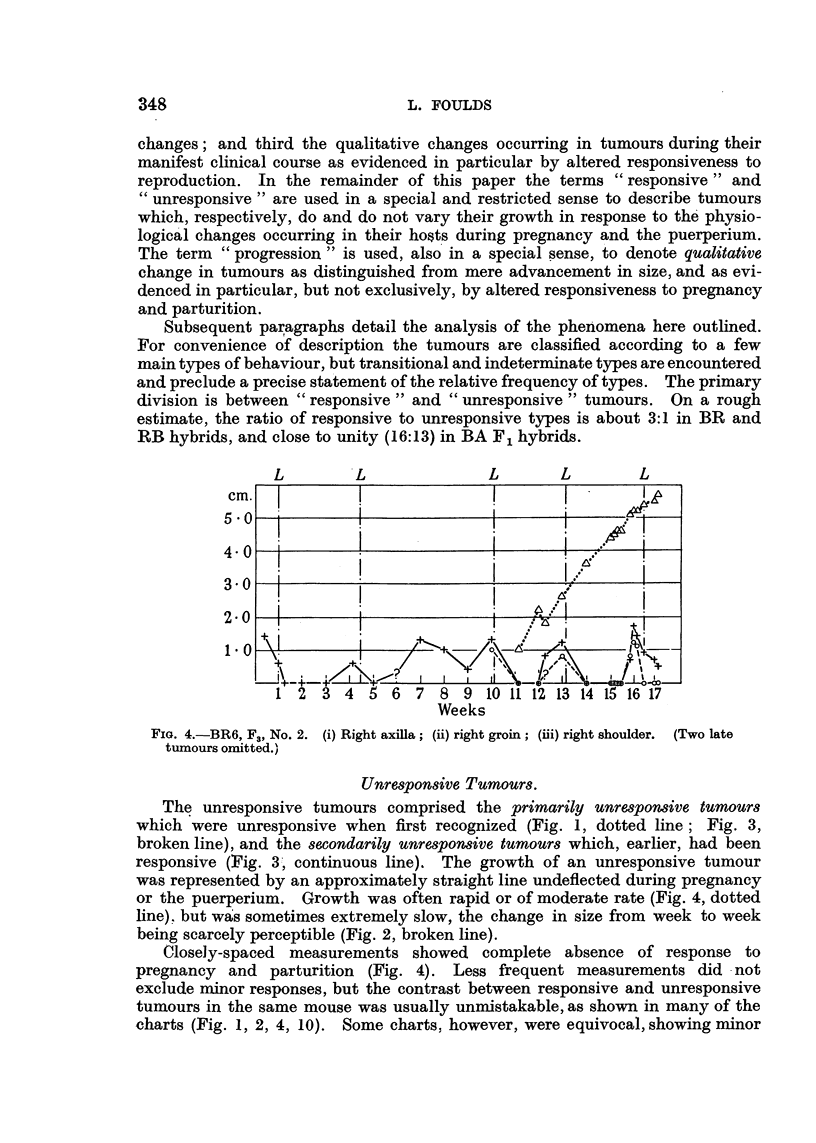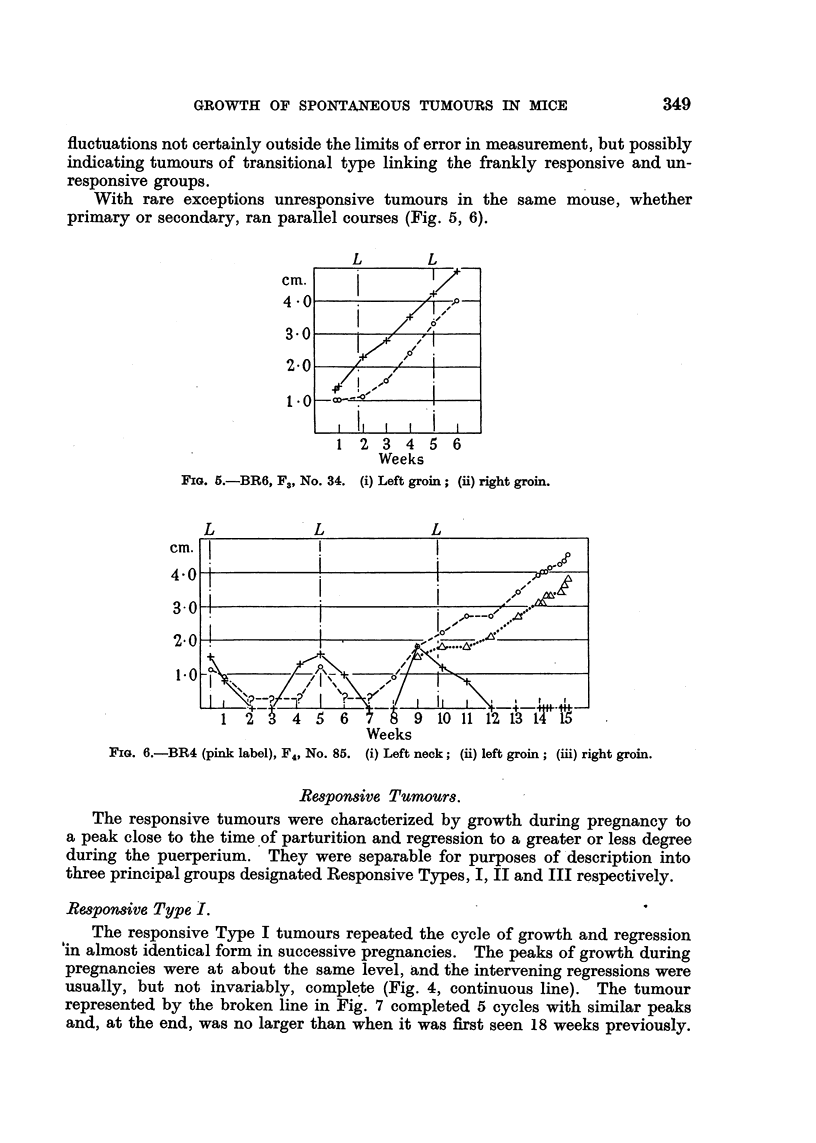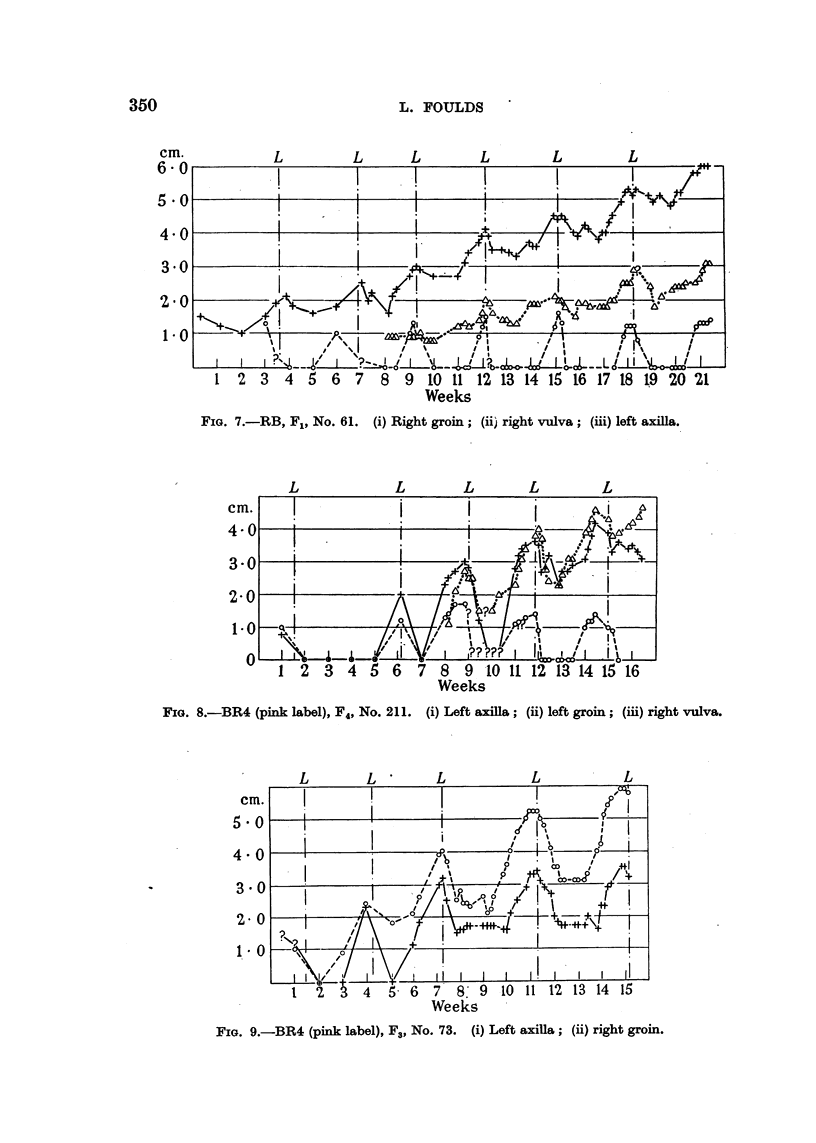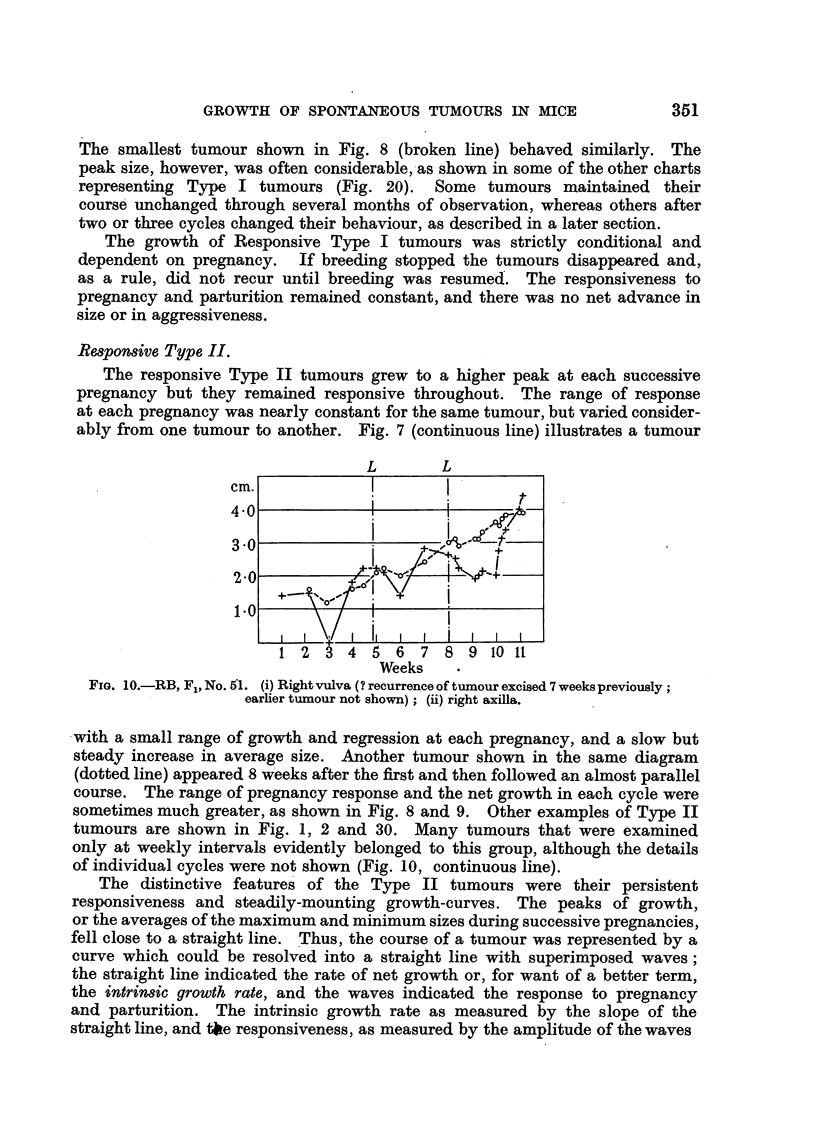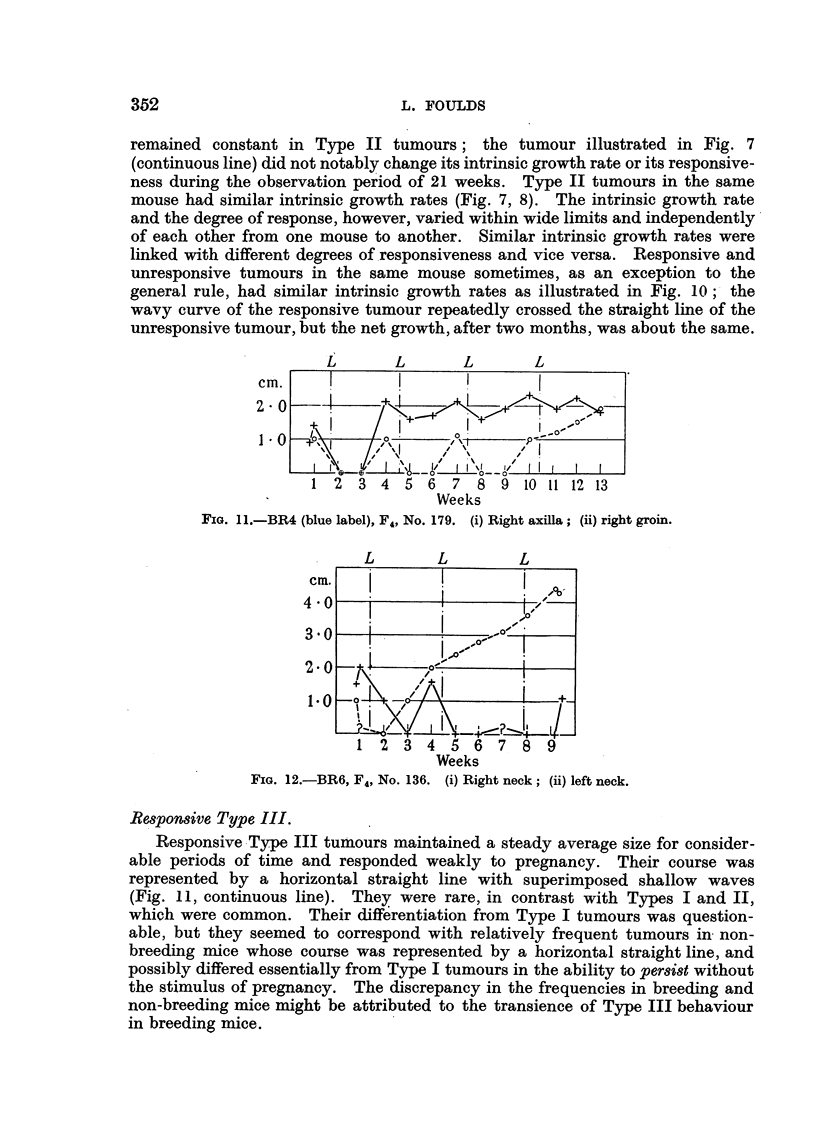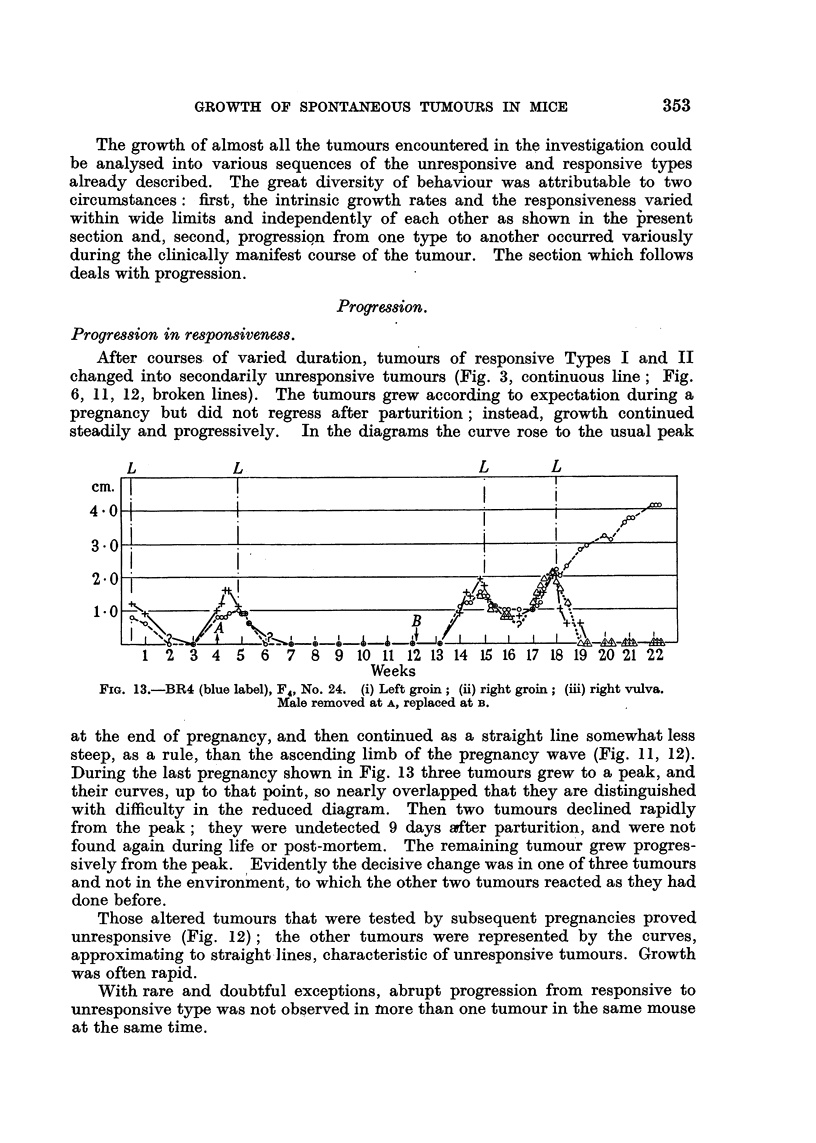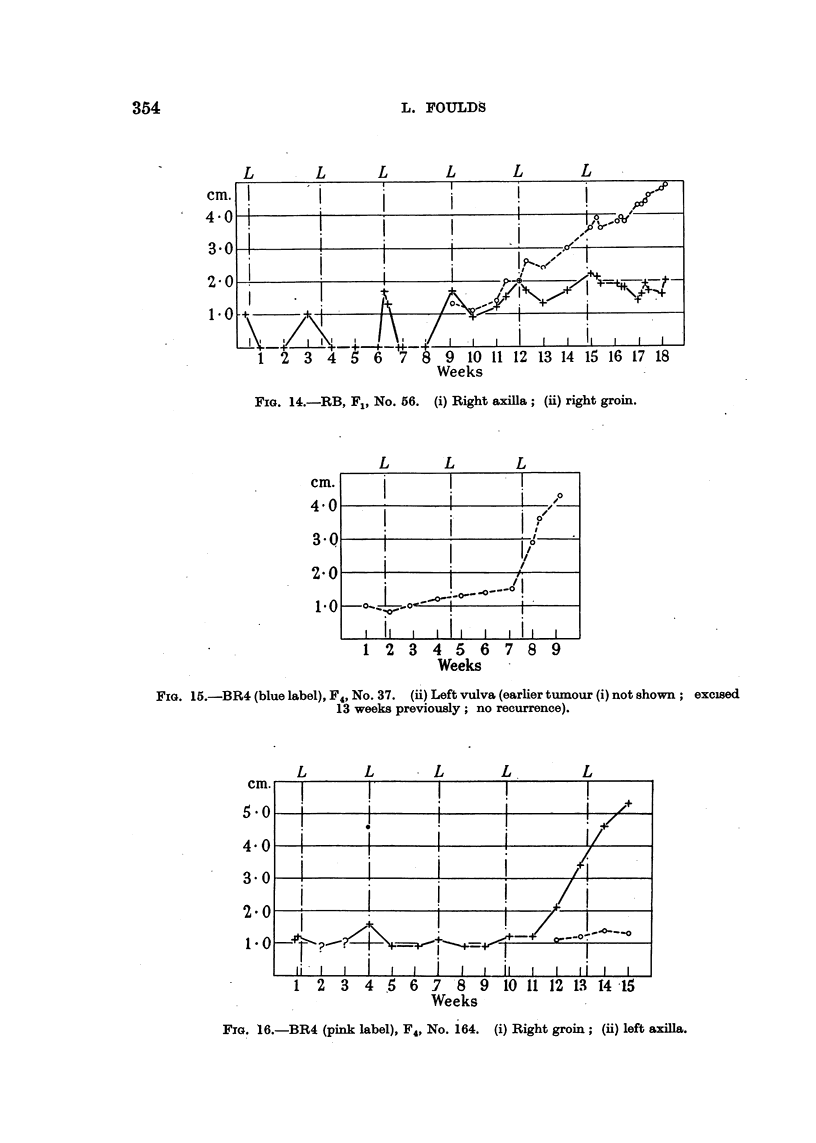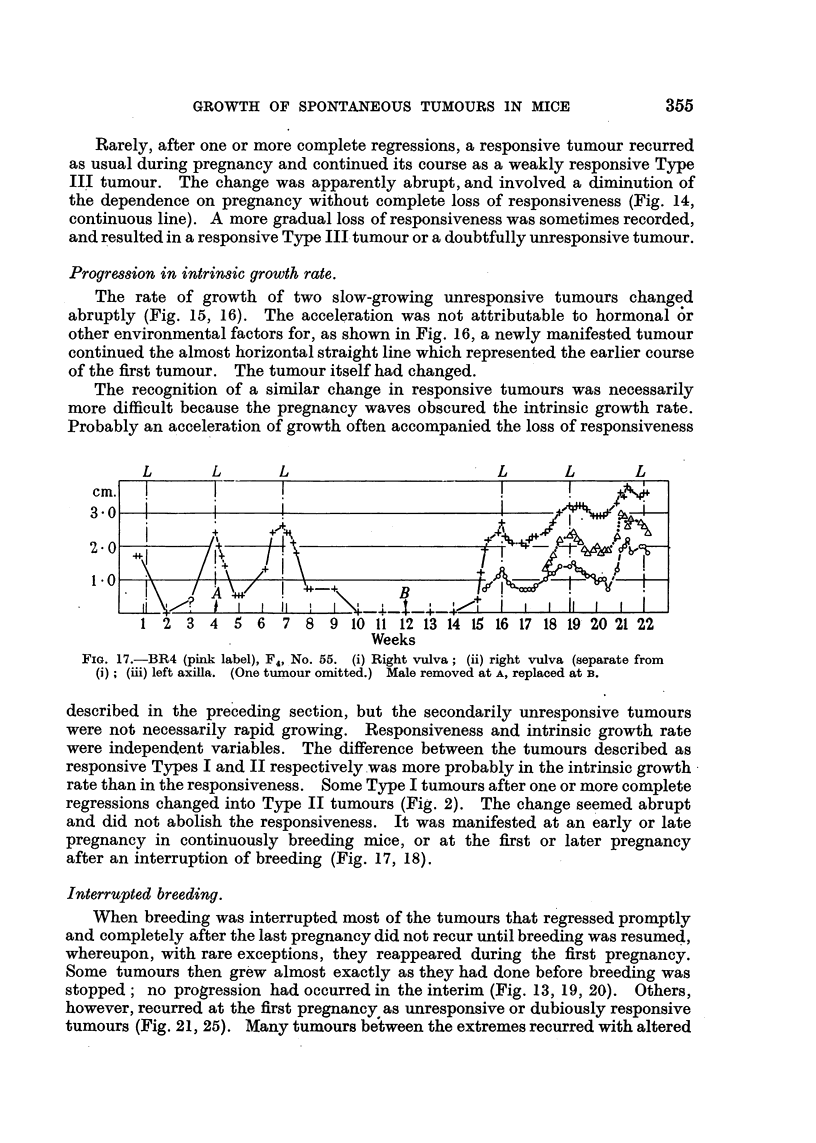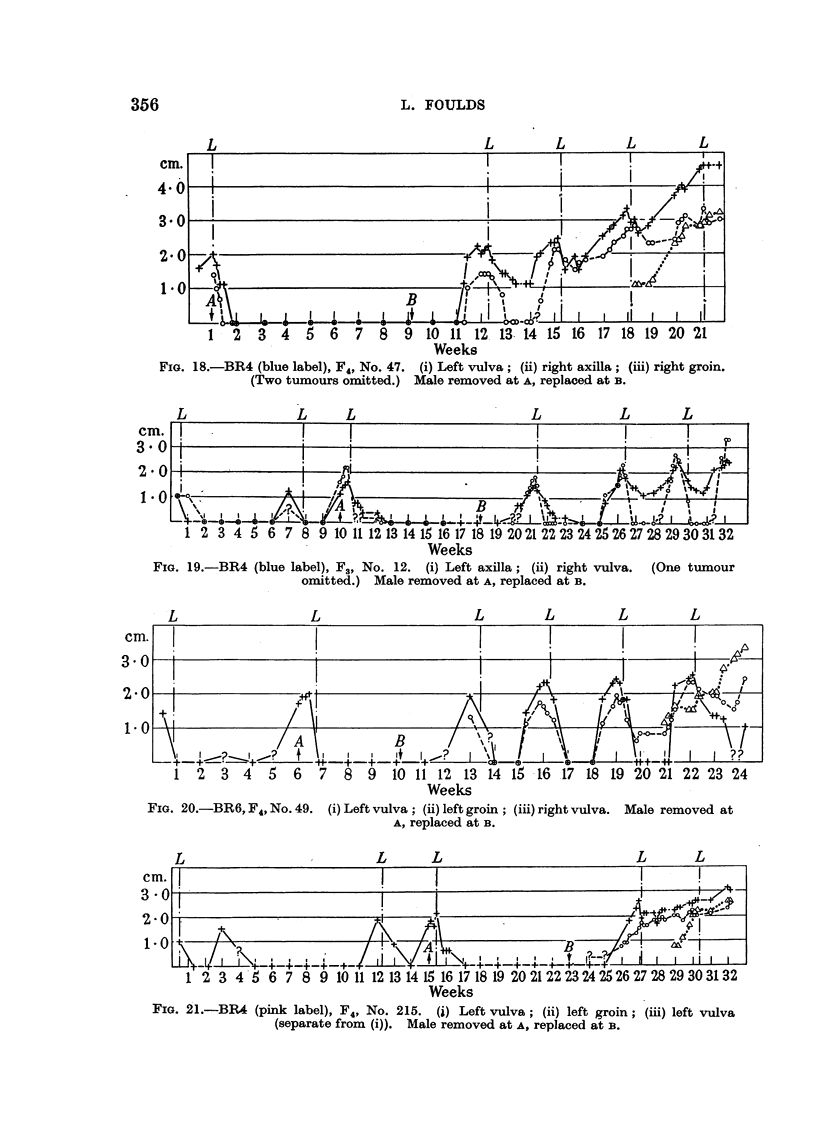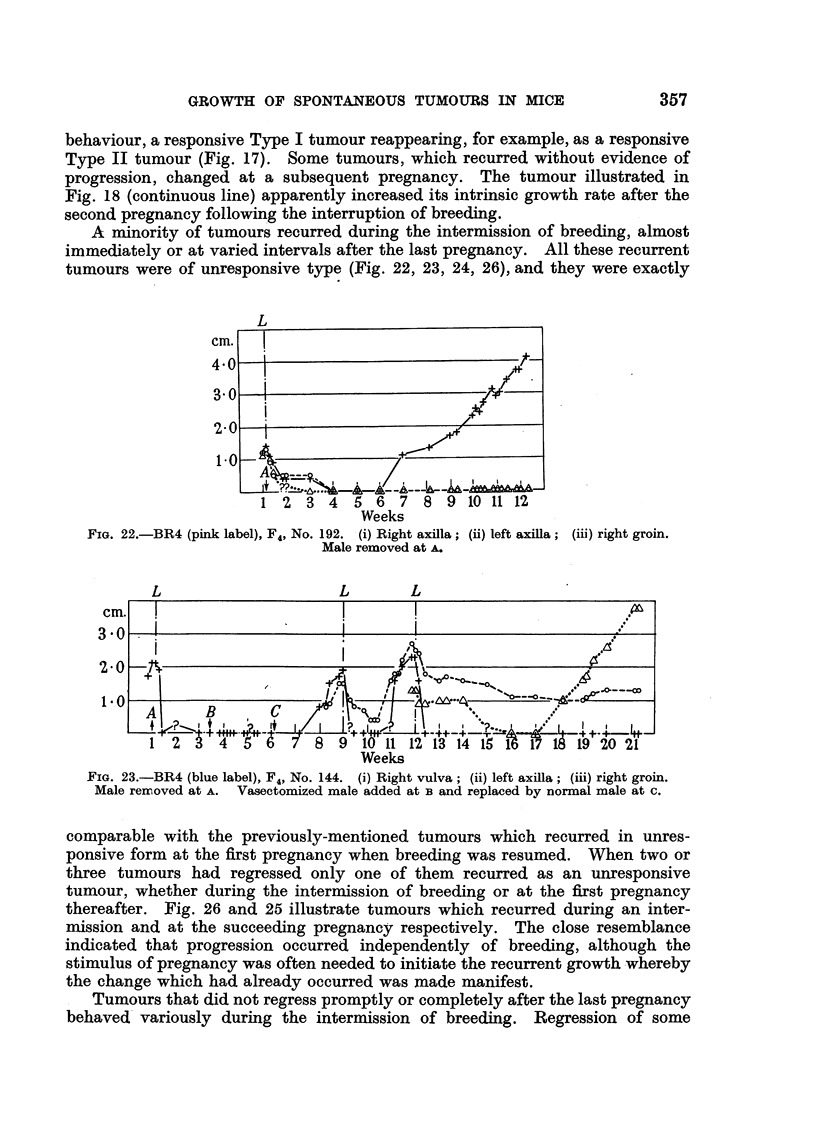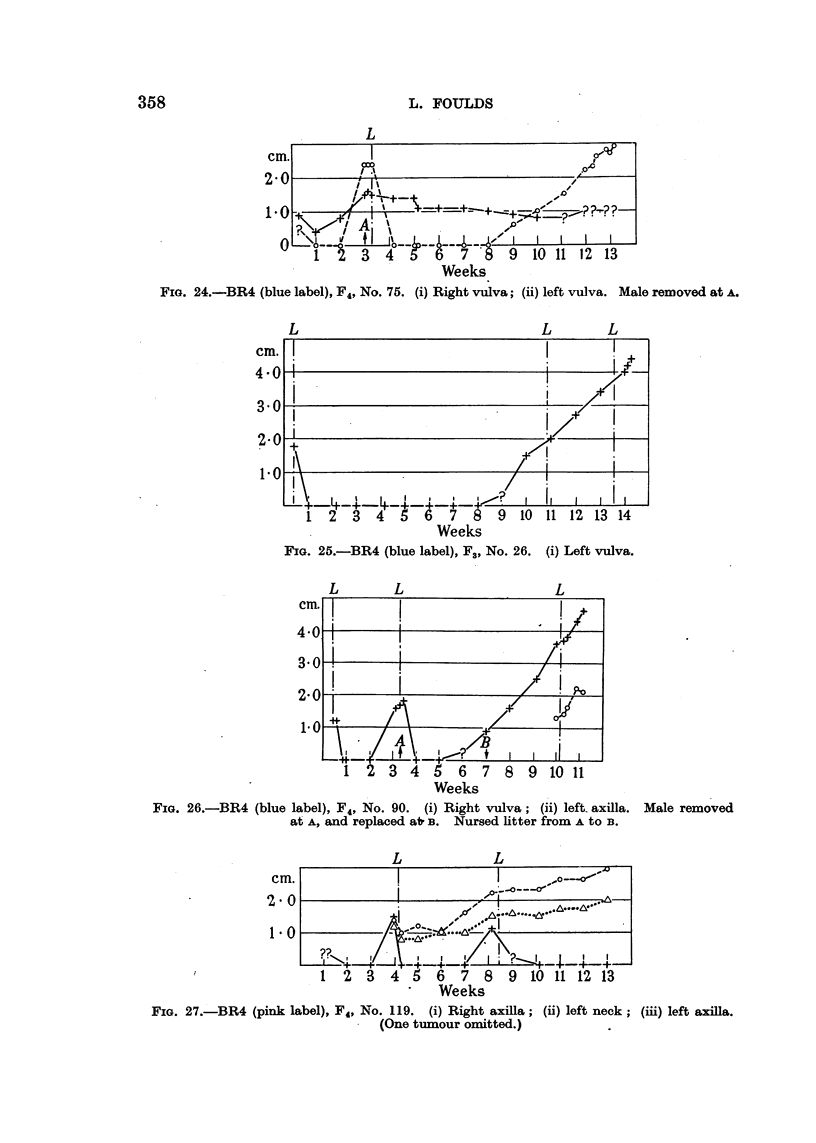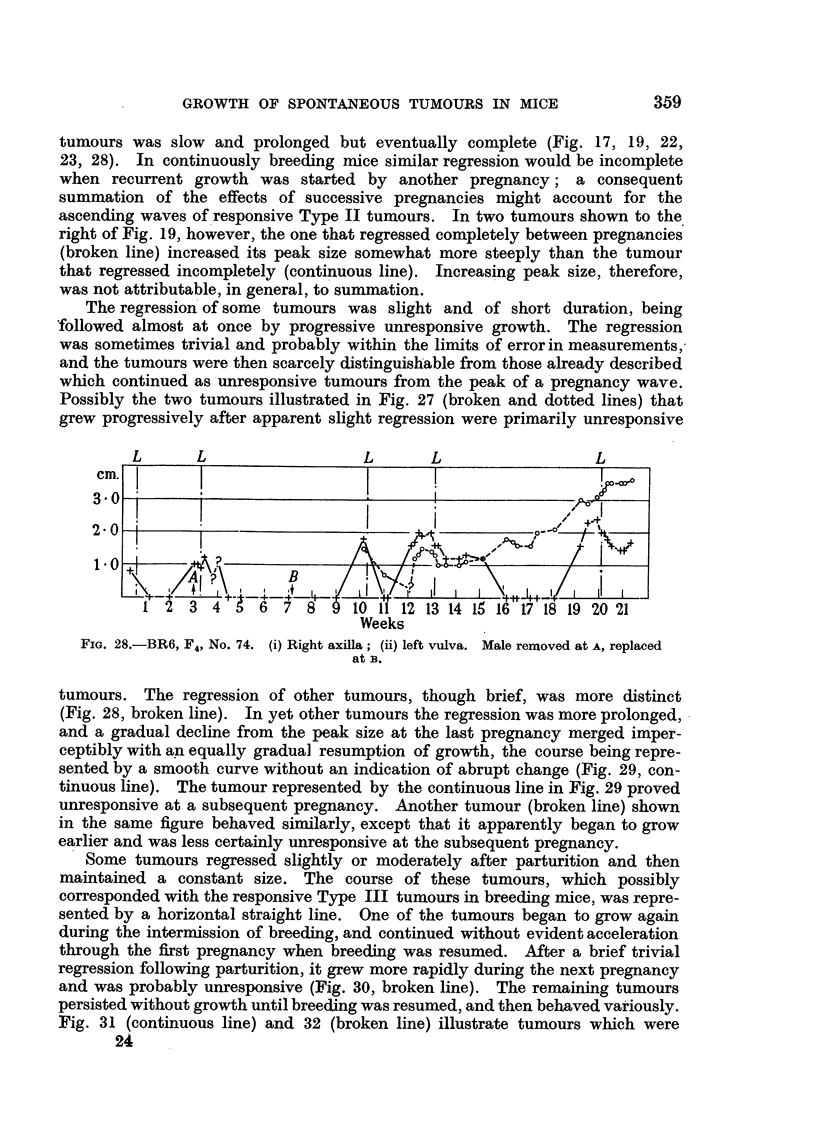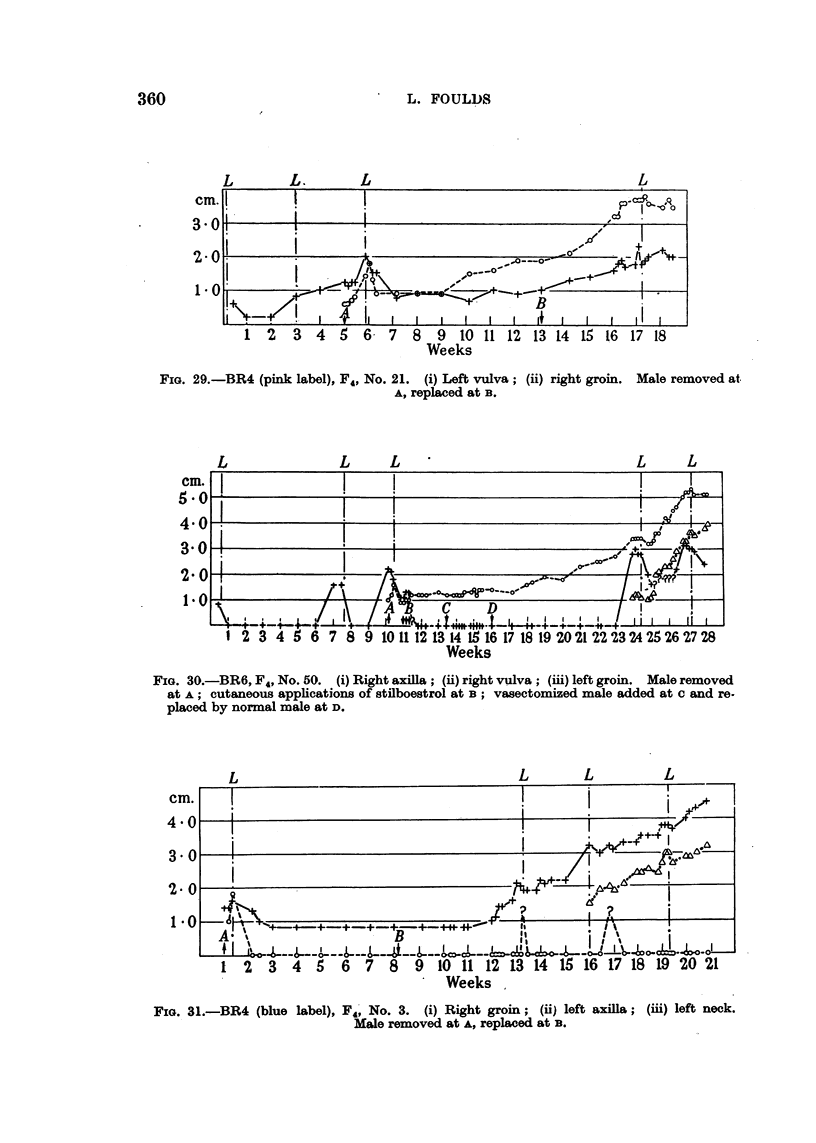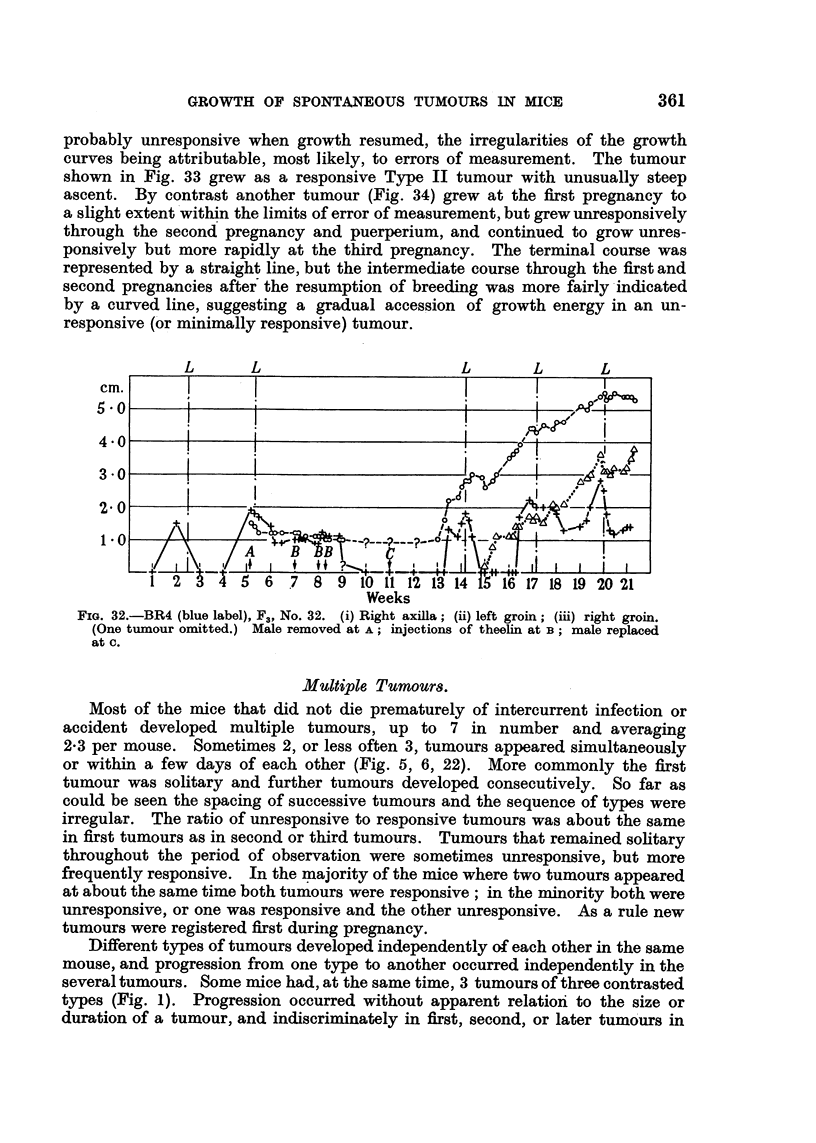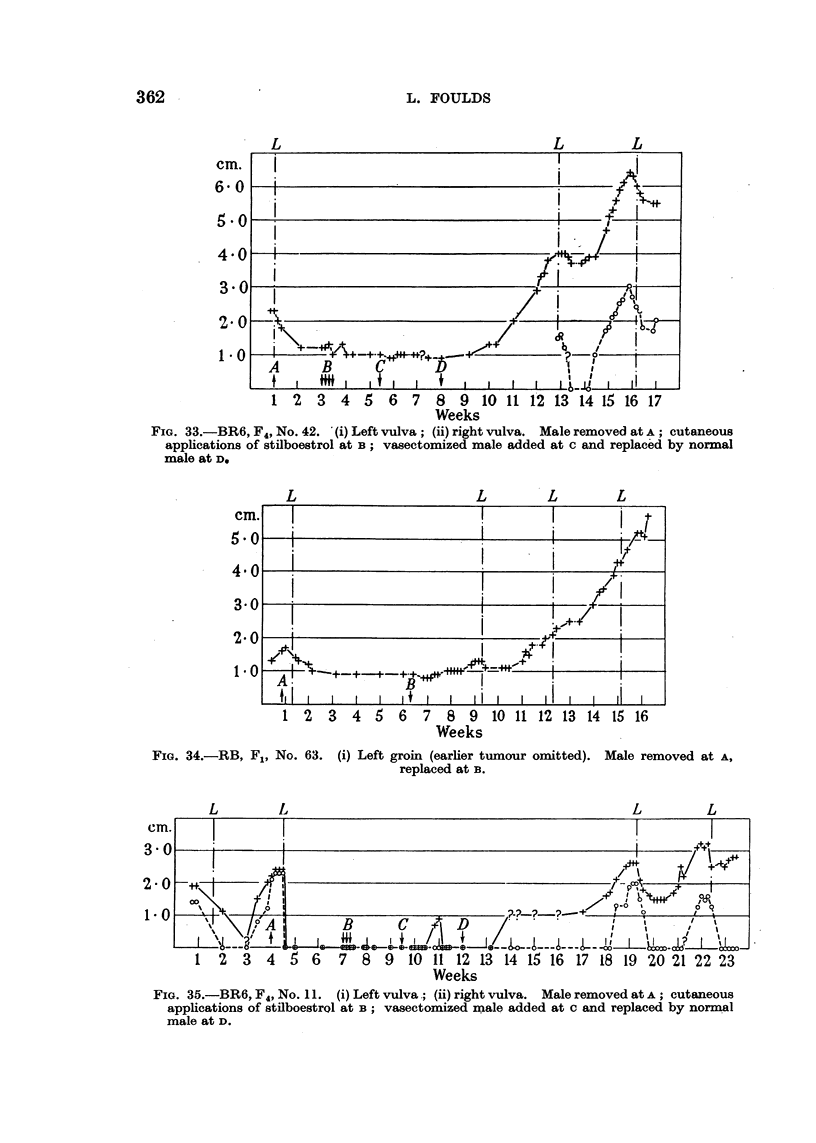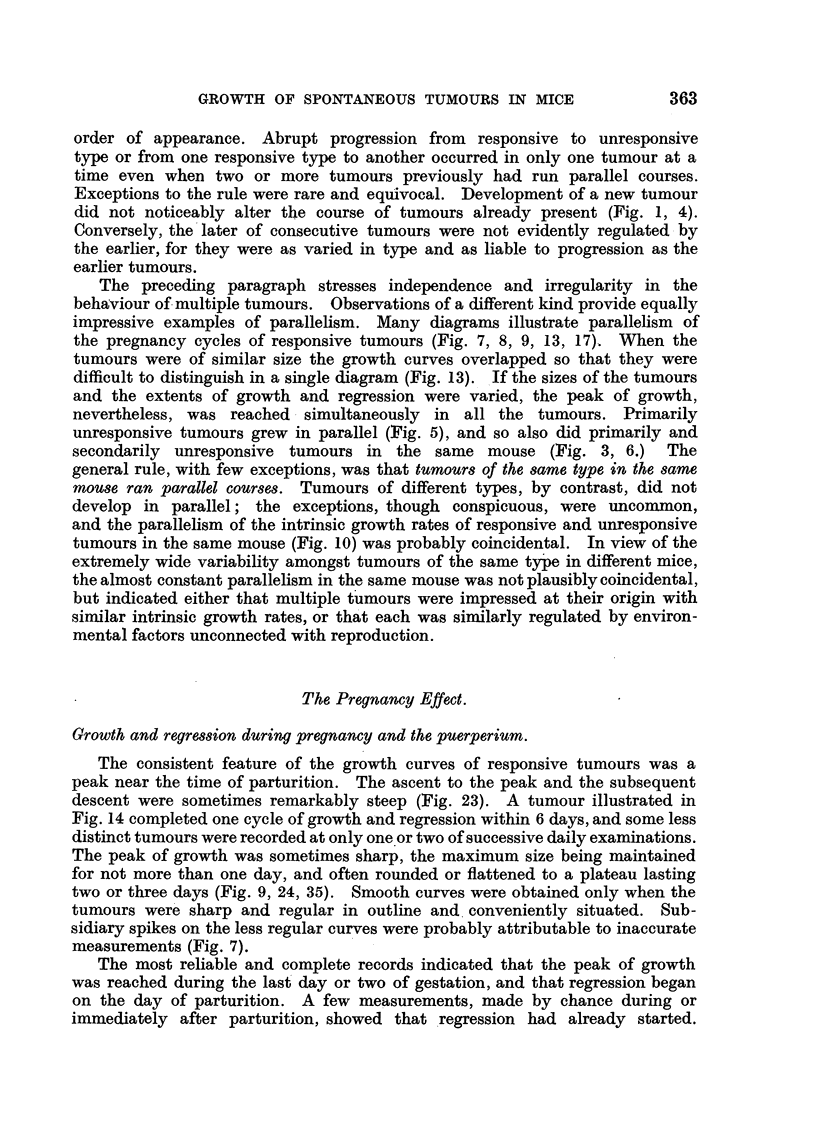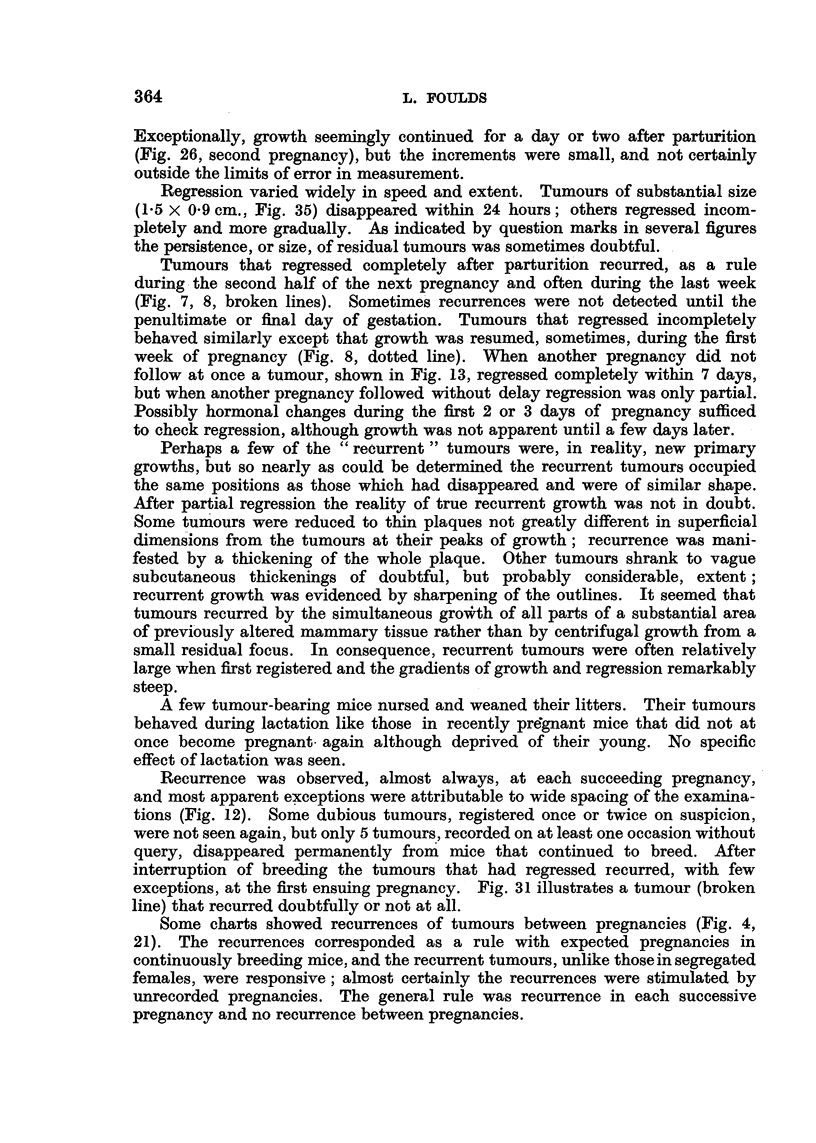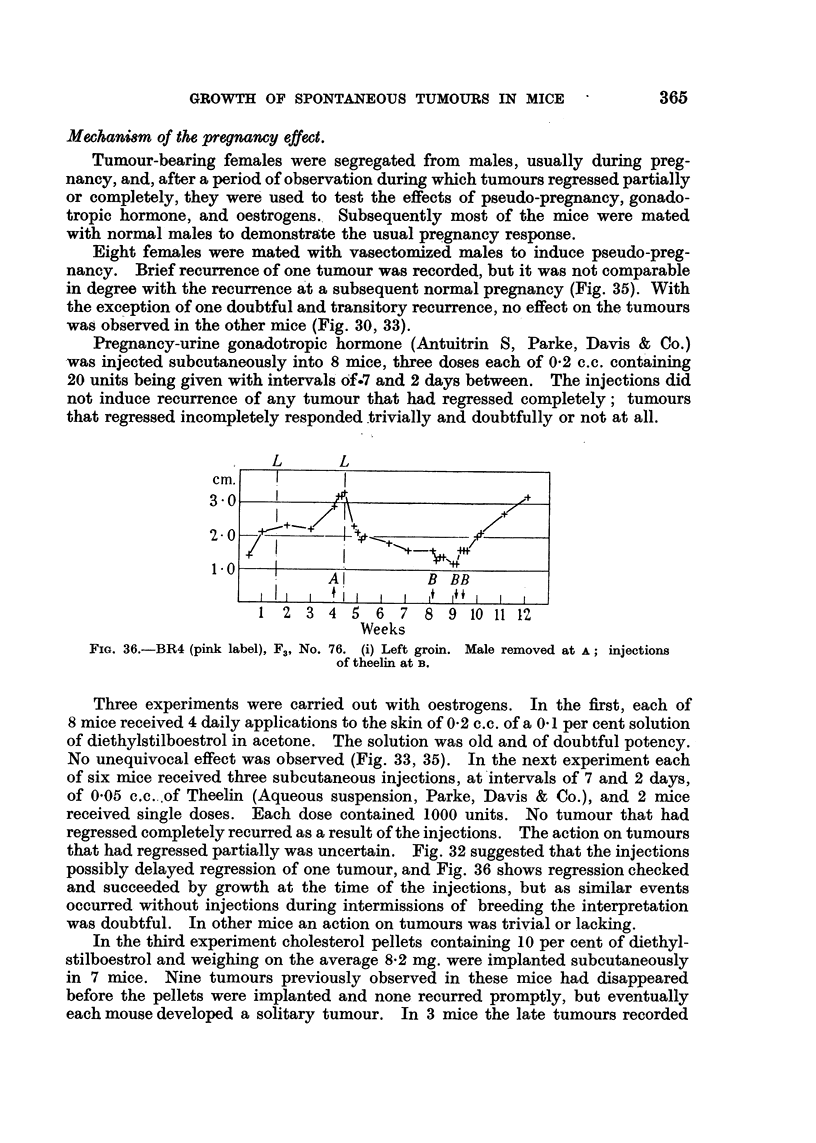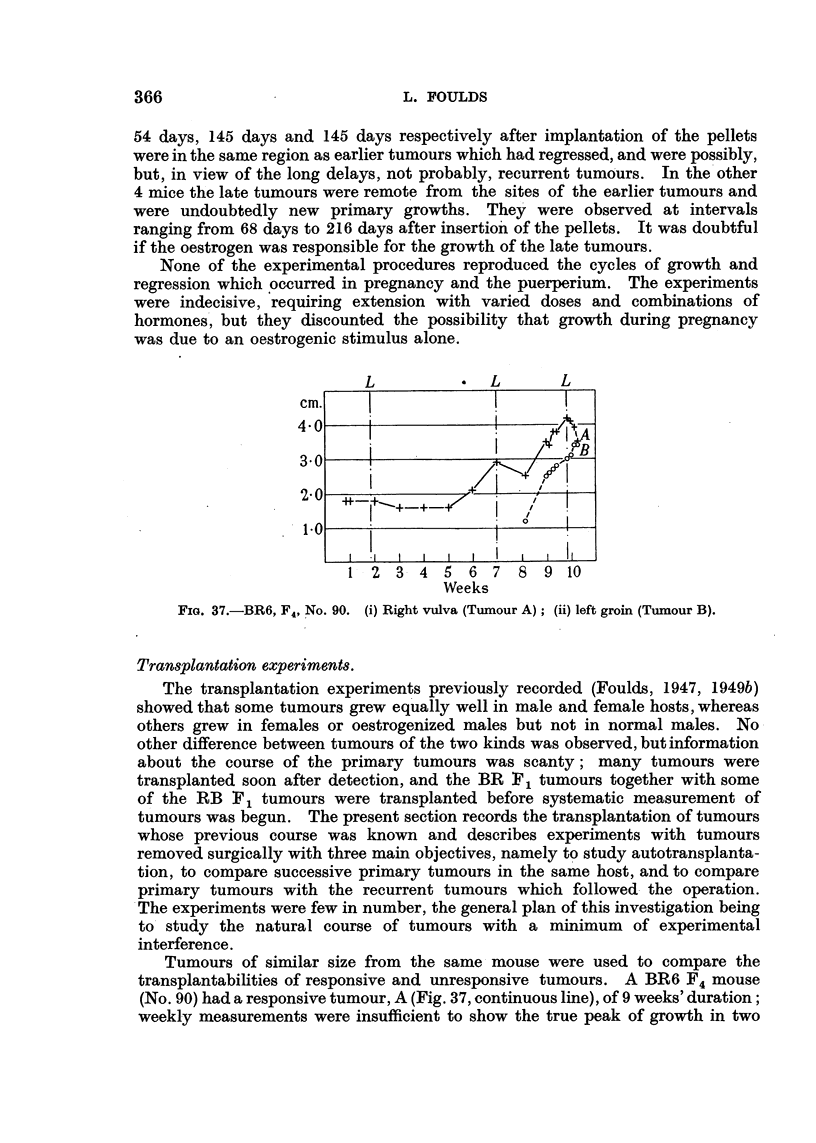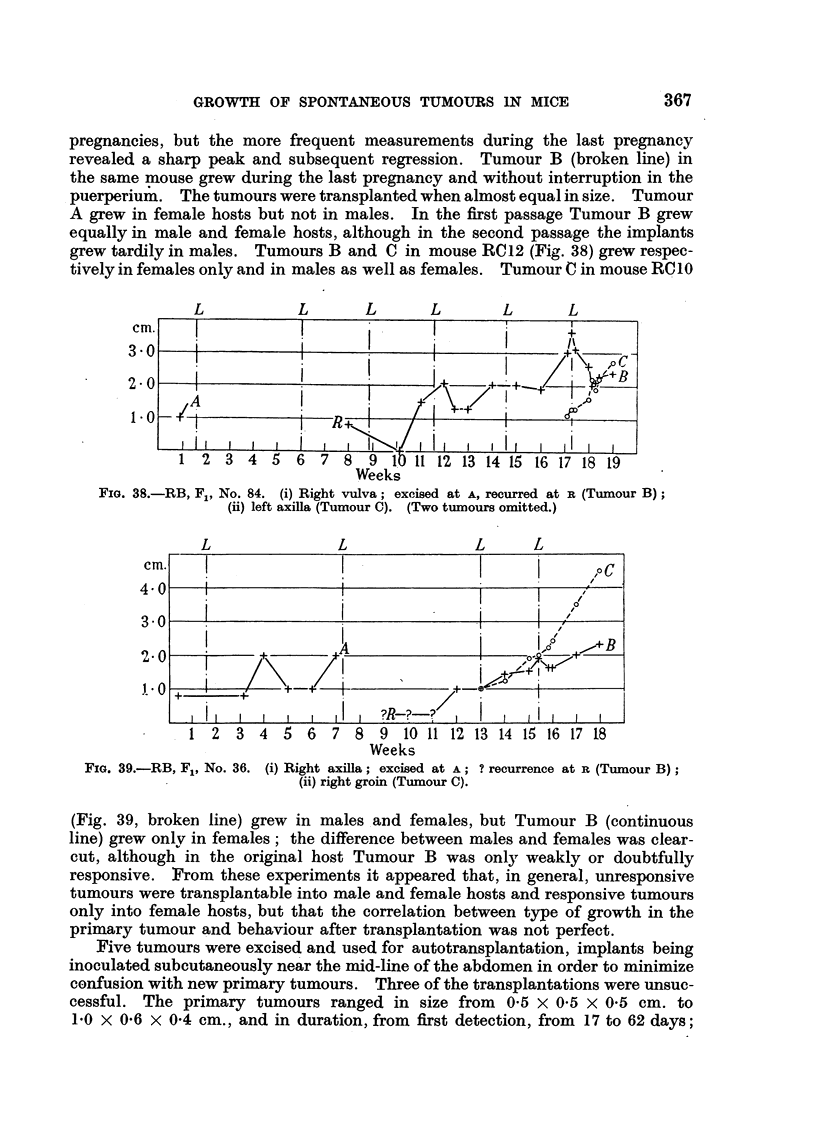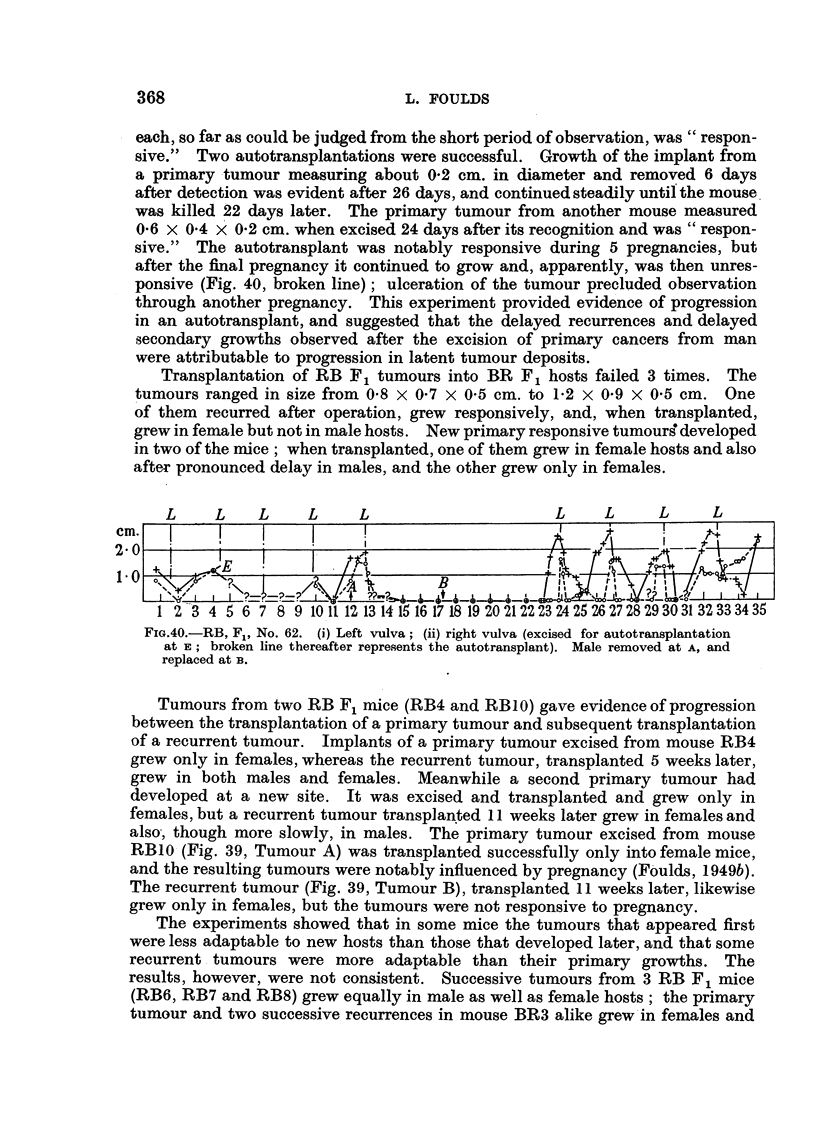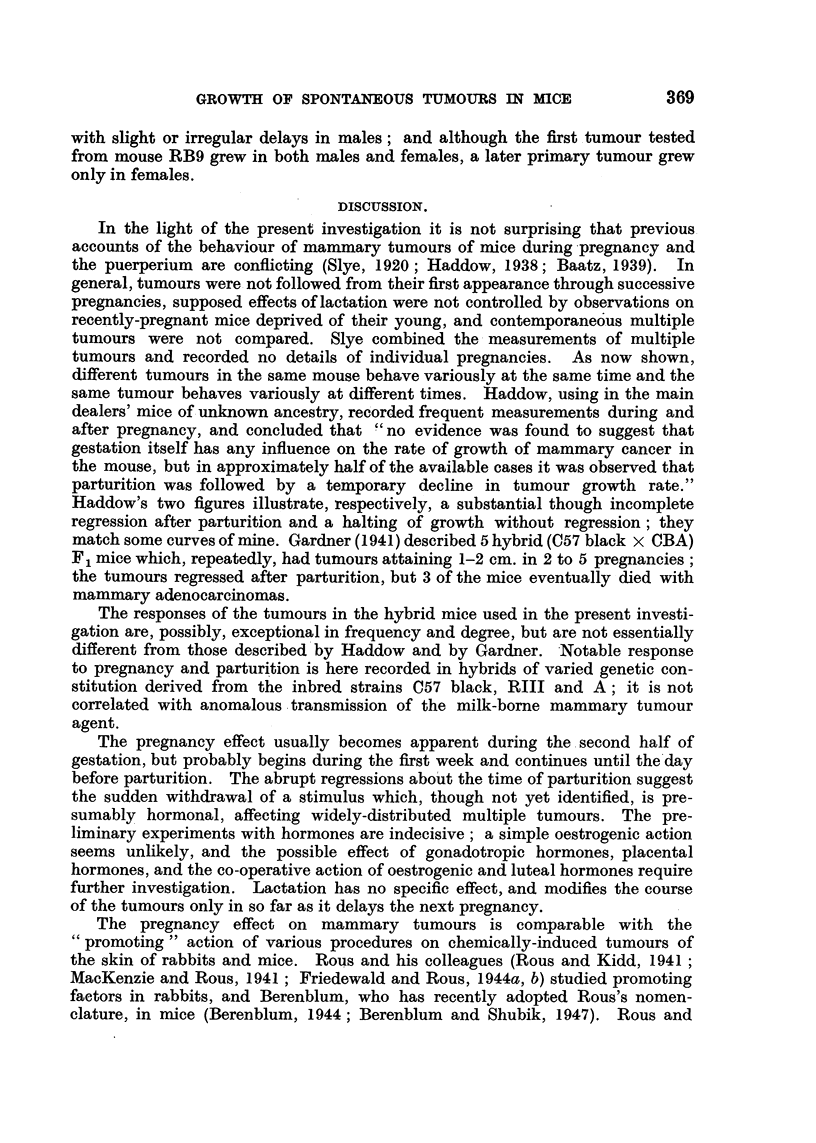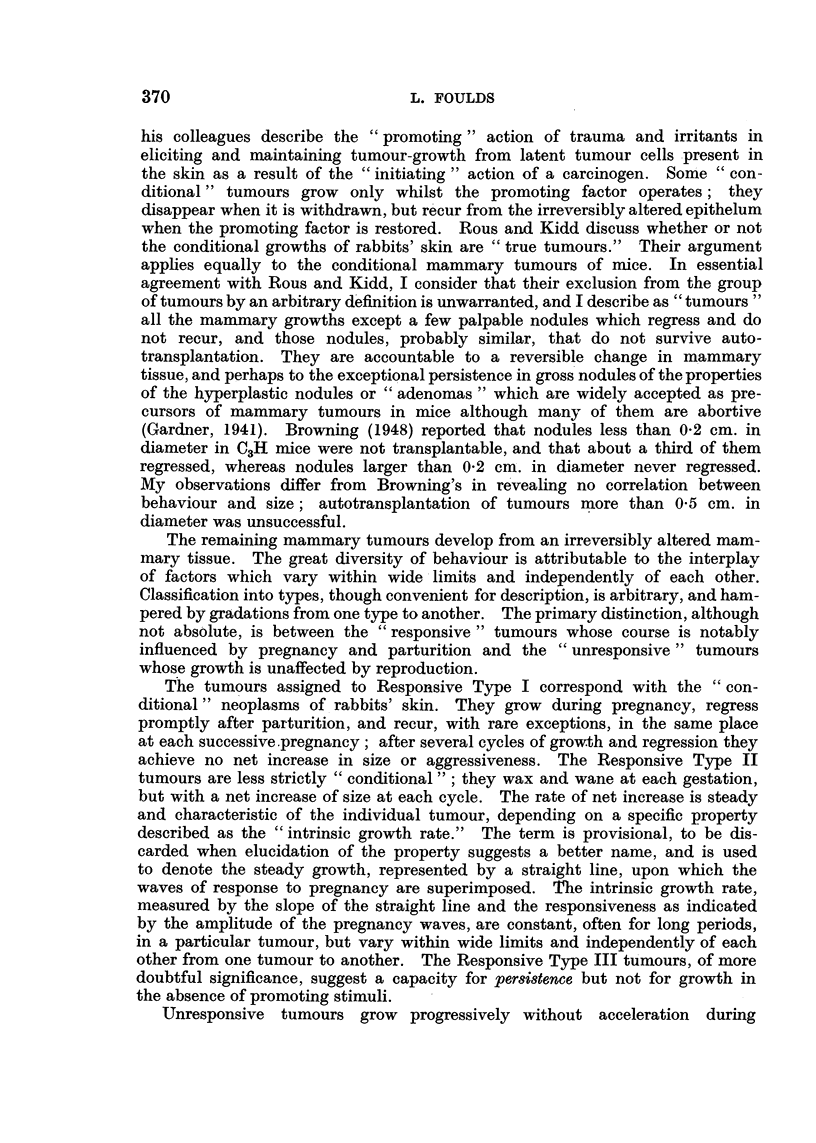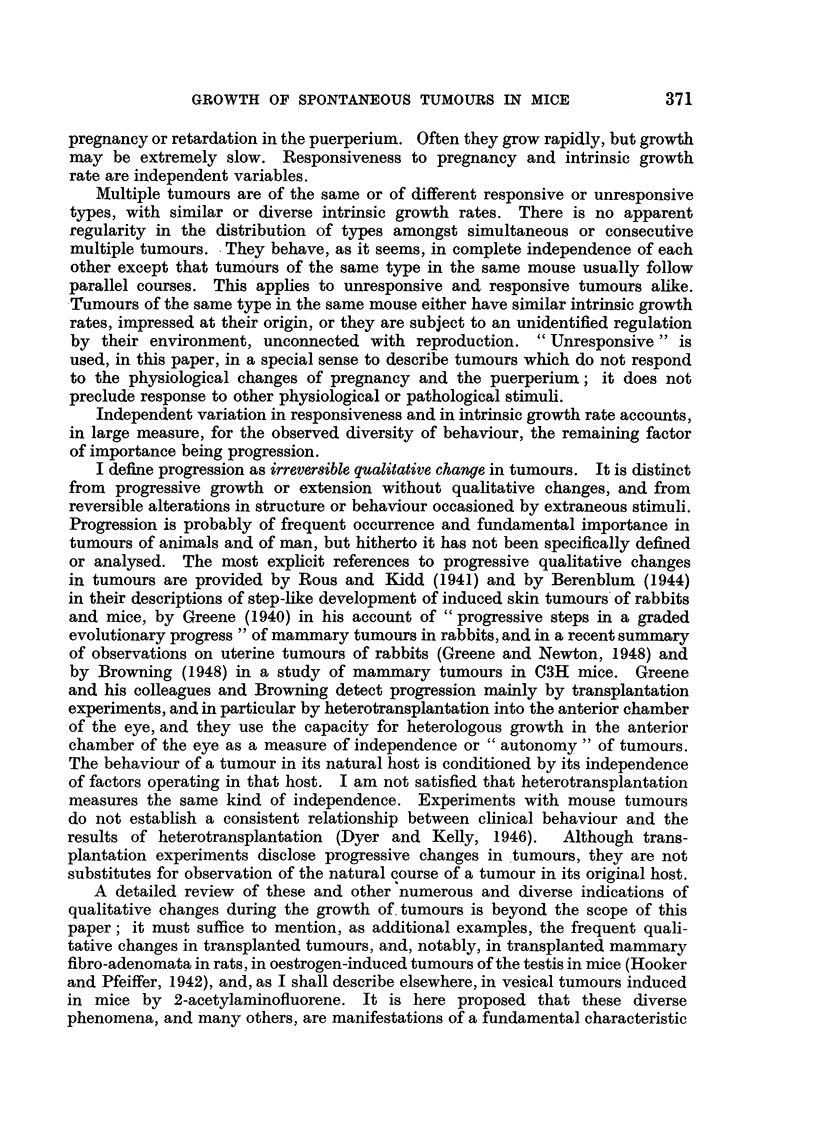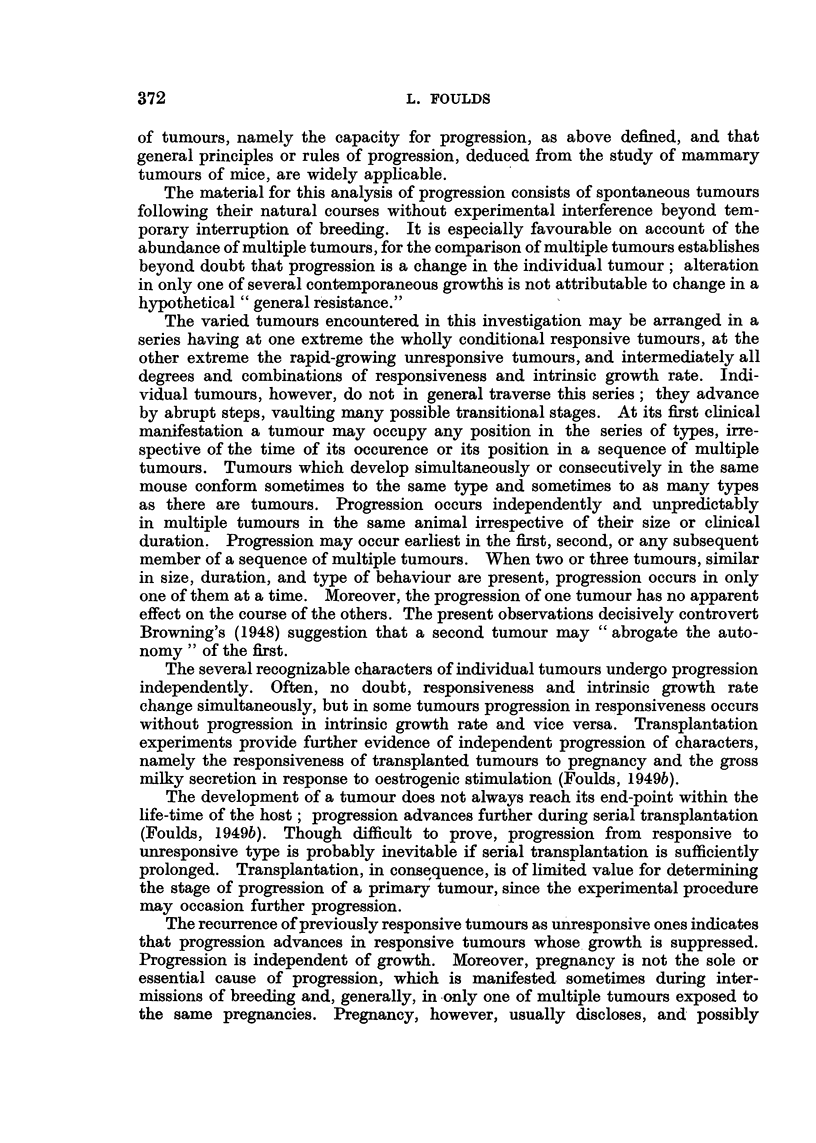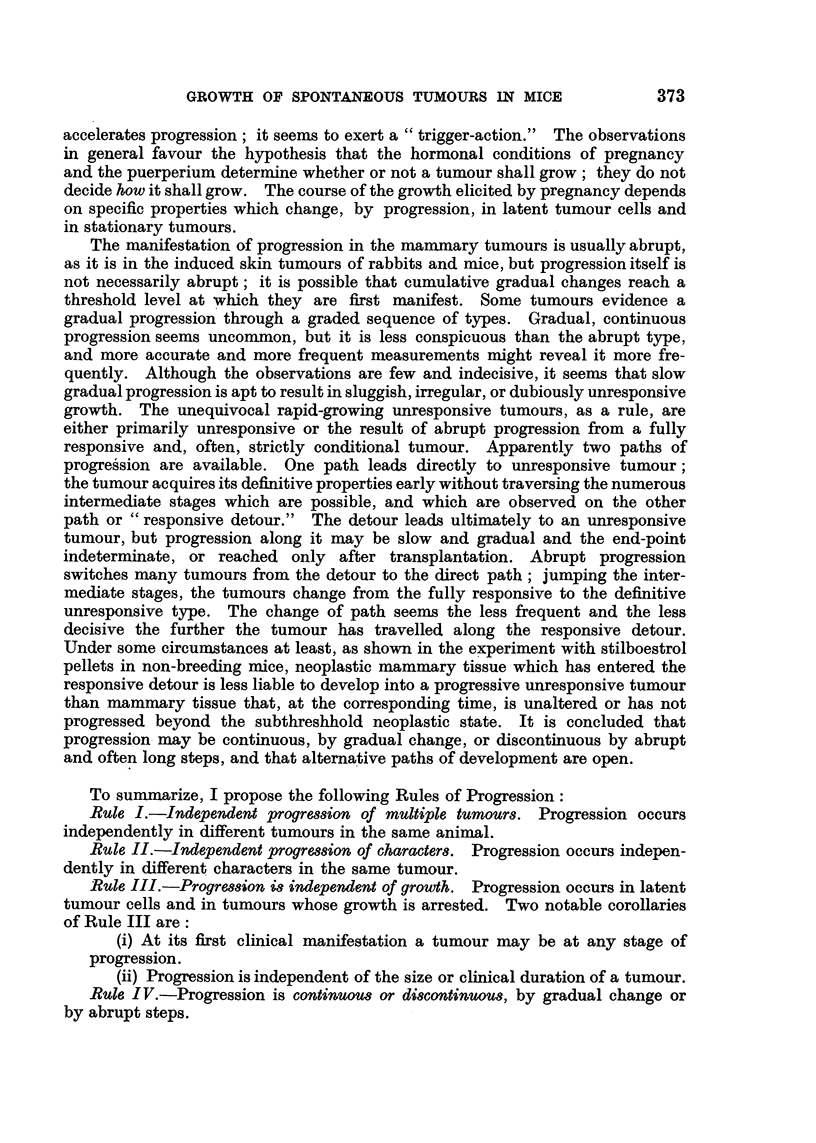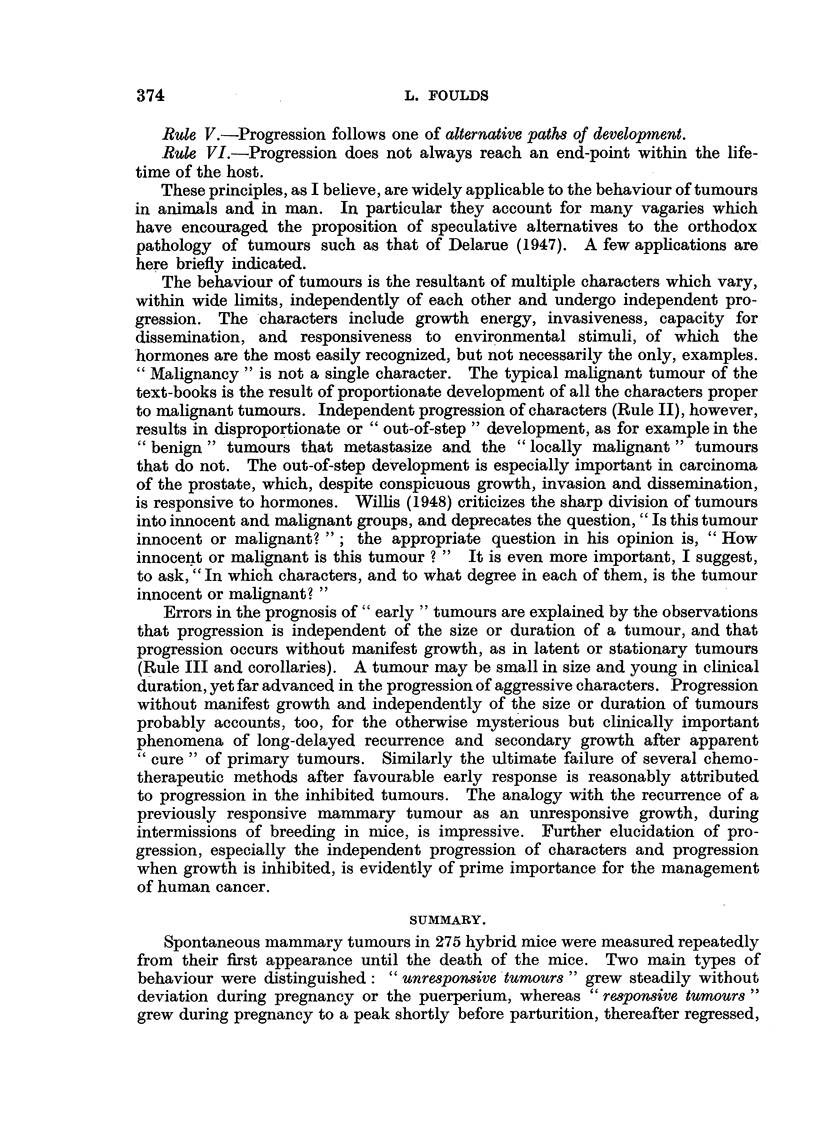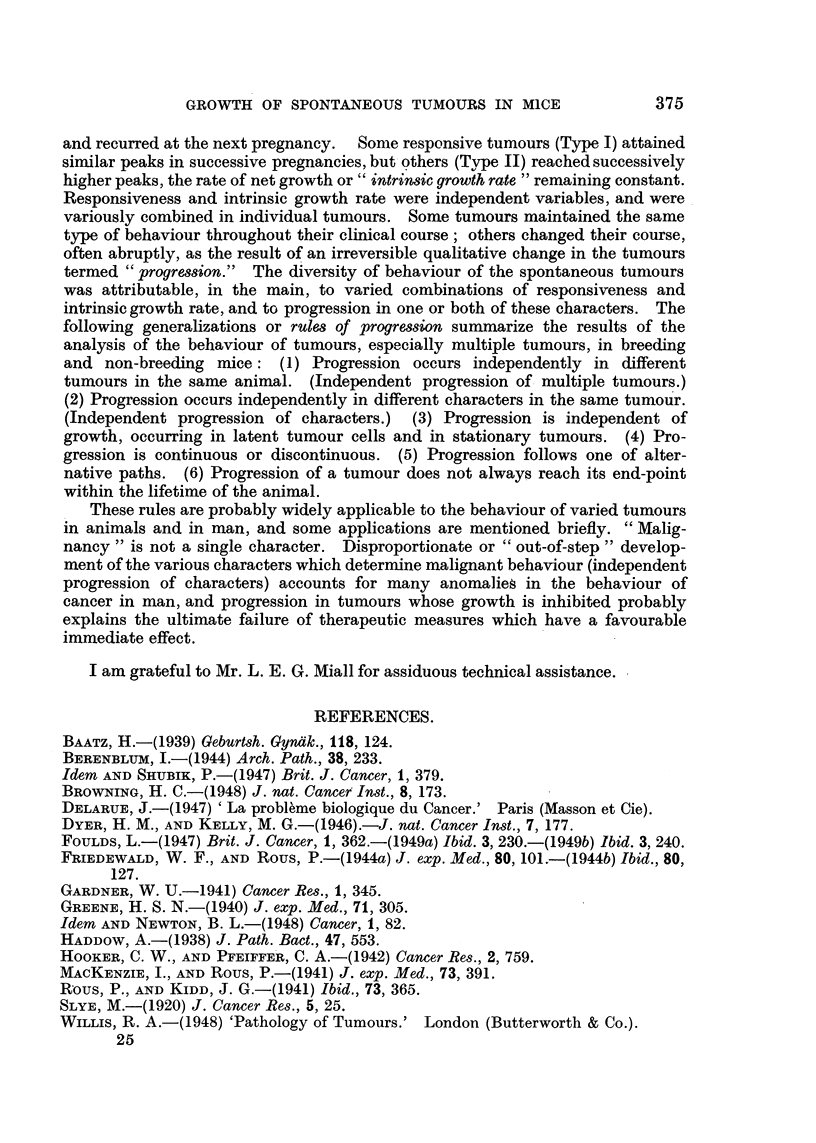# Mammary Tumours in Hybrid Mice: Growth and Progression of Spontaneous Tumours

**DOI:** 10.1038/bjc.1949.40

**Published:** 1949-09

**Authors:** L. Foulds


### MAMMARY TUMOURS IN HYBRID MICE: GROWTH AND PROGRESSION OF SPONTANEOUS TUMOURS.

L. FOULDS.

*From the Laboratories of the Imperial Cancer Research Fund, London, N.W.7.*

Received for publication March 14, 1949.

PREVIOUS observations (Foulds, 1947, 1949*b*) showed that the growth of some transplanted mammary tumours in hybrid mice depended on a hormonal stimulus which operated in normal female mice but not in normal males; artificially-administered oestrogen supplied the necessary stimulus for growth in males. Some of the tumours were independent of the hormonal stimulus at the first transplantation; others lost their dependence after several transfers. Dependence on hormones seemed to characterize one stage in the life-history of mammary tumours in hybrid mice. The present paper deals with spontaneous mammary tumours growing in their original hosts; it records cycles of growth and regression of tumours in successive pregnancies, and a progression of the tumours towards independence of hormonal stimuli.

### MATERIAL AND METHODS

The investigation comprised repeated measurements of tumours in 275 mice which carried, before death, a total of 655 mammary growths. The majority of the mice were BR hybrids, mostly of the $F_3$ and $F_4$ generations, and made up of 71 mice of strain BR4 (pink label), 90 of strain BR4 (blue label), and 69 of strain BR6. The remaining mice were 28 RB $F_1$ hybrids and 17 BA $F_1$ hybrids. A preceding paper (Foulds, 1949*a*) describes the source of these hybrids and the incidence of tumours in them.

The mice were subjected to forced breeding and most of them had pregnancies in rapid succession throughout the period of observation, even when bearing multiple large tumours. Some intermissions of breeding were attributable to temporary absence of males or to infertility of the males.

The mice were inspected twice weekly. Tumours were recognized usually when about 0·5 cm. in diameter, and weekly thereafter two main diameters were measured with calipers. Growth charts were made by plotting the sum of the two diameters against time. Post-mortem measurements usually agreed satisfactorily with those made during life; measurements on regressing tumours were the most difficult and subject to the largest errors. Frequent, often daily, measurements were made on selected mice.

Litters were recorded each morning except on Sundays and public holidays. The time of parturition as shown on the charts was usually later than the actual time by unknown periods of up to 24 hours on weekdays and 48 hours at week-ends. A more accurate timing was not attempted systematically, but was sometimes achieved, by chance, when mice were examined during parturition.

Some litters, especially those promptly devoured by their mothers, were not recorded, and others, probably, were attributed to the wrong mothers. These errors were minimized in selected groups of mice examined frequently during pregnancy. Interruption of breeding was ensured in other groups by segregation of the females.

Some mice died, but most were killed when they appeared ill, when ulceration was present or threatening, or when large tumours seemed unlikely to yield additional information. The mice were examined post-mortem except for a few that were eaten ; the tumours were measured and pieces of them fixed for histological examination.

In the charts the first tumour, or largest of contemporaneous tumours, is represented by a continuous line joining crosses which indicate the actual measurements ; the second tumour is represented by a broken line and circles, and the third by a dotted line and triangles. To avoid overcrowding of the diagrams, tumours beyond the third are omitted from the reproductions. Vertical broken lines, headed " L," show the recorded litter-dates. The charts are reduced to approximately the same extent for reproduction except for Fig. 19, 21, 30, 40 covering especially long periods of observation, which are reduced to a greater degree. Scales of time and size are shown on each chart. The legends give the situations of the tumours which are numbered (i) continuous line, (ii) broken line and (iii) dotted line.

Fig. 1.—BR6, $F_4$, No. 175. (i) Right axilla ; (ii) left groin ; (iii) right neck.

RESULTS.

*General Features of Growth.*

Some tumours grew steadily from their first appearance until the death of their hosts ; as described by Haddow (1938), their course was represented on the charts made by plotting the sum of 2 diameters against time by an approximately straight line (Fig. 1, dotted line). Other tumours, constituting a majority, were conspicuously different. Discovered, as a rule, during pregnancy, they disappeared after parturition, recurred during the next pregnancy, grew to a peak at or near the time of parturition and thereafter regressed again, partially or completely. Some tumours repeated the cycles of growth and regression in almost identical form in several successive pregnancies (Fig. 1, broken line).

Others grew to a higher peak in each succeeding pregnancy, the charts showing ascending curves with peaks near the times of parturition and intervening troughs (Fig. 1, continuous line). Tumours sometimes grew in that way throughout their course. Many tumours, however, changed their course conspicuously, and apparently abruptly, during the period of observation; after several cycles of growth and regression with equal peaks they continued in cycles with steadily-ascending peaks (Fig. 2, continuous line) or, after pronounced waves of growth and regression in several pregnancies, they grew progressively without acceleration at succeeding pregnancies and without regression between pregnancies, their new course being represented on the charts by a straight line (Fig. 3, continuous line).

FIG. 2.—BR4 (blue label), $F_4$, No. 145. (i) Right groin; (ii) right vulva; (iii) left neck.

FIG. 3.—BR6, $F_4$, No. 178. (i) Left axilla; (ii) right axilla.

In most of the mice from 1 to 6 new tumours appeared during the course of the first. The second and subsequent tumours were as varied in type as those which appeared first. The several tumours in the same mouse did not conform to a single type of growth nor to a regular sequence of types. Fig. 1 shows three different types of behaviour at the same time in one mouse.

At least three major factors contributed to the wide diversity of behaviour amongst spontaneous tumours, and even amongst multiple tumours in the same mouse: first, the regulation of tumour growth by the environmental, and presumably hormonal, changes occurring during pregnancy and the puerperium; second, the specific reactivities of particular tumours to the environmental changes; and third the qualitative changes occurring in tumours during their manifest clinical course as evidenced in particular by altered responsiveness to reproduction. In the remainder of this paper the terms "responsive" and "unresponsive" are used in a special and restricted sense to describe tumours which, respectively, do and do not vary their growth in response to the physiological changes occurring in their hosts during pregnancy and the puerperium. The term "progression" is used, also in a special sense, to denote *qualitative* change in tumours as distinguished from mere advancement in size, and as evidenced in particular, but not exclusively, by altered responsiveness to pregnancy and parturition.

Subsequent paragraphs detail the analysis of the phenomena here outlined. For convenience of description the tumours are classified according to a few main types of behaviour, but transitional and indeterminate types are encountered and preclude a precise statement of the relative frequency of types. The primary division is between "responsive" and "unresponsive" tumours. On a rough estimate, the ratio of responsive to unresponsive types is about 3:1 in BR and RB hybrids, and close to unity (16:13) in BA $F_1$ hybrids.

FIG. 4.—BR6, $F_3$, No. 2. (i) Right axilla; (ii) right groin; (iii) right shoulder. (Two late tumours omitted.)

*Unresponsive Tumours.*

The unresponsive tumours comprised the *primarily unresponsive tumours* which were unresponsive when first recognized (Fig. 1, dotted line; Fig. 3, broken line), and the *secondarily unresponsive tumours* which, earlier, had been responsive (Fig. 3, continuous line). The growth of an unresponsive tumour was represented by an approximately straight line undeflected during pregnancy or the puerperium. Growth was often rapid or of moderate rate (Fig. 4, dotted line), but was sometimes extremely slow, the change in size from week to week being scarcely perceptible (Fig. 2, broken line).

Closely-spaced measurements showed complete absence of response to pregnancy and parturition (Fig. 4). Less frequent measurements did not exclude minor responses, but the contrast between responsive and unresponsive tumours in the same mouse was usually unmistakable, as shown in many of the charts (Fig. 1, 2, 4, 10). Some charts, however, were equivocal, showing minor fluctuations not certainly outside the limits of error in measurement, but possibly indicating tumours of transitional type linking the frankly responsive and unresponsive groups.

With rare exceptions unresponsive tumours in the same mouse, whether primary or secondary, ran parallel courses (Fig. 5, 6).

FIG. 5.—BR6, $F_3$, No. 34. (i) Left groin ; (ii) right groin.

FIG. 6.—BR4 (pink label), $F_4$, No. 85. (i) Left neck ; (ii) left groin ; (iii) right groin.

### *Responsive Tumours.*

The responsive tumours were characterized by growth during pregnancy to a peak close to the time of parturition and regression to a greater or less degree during the puerperium. They were separable for purposes of description into three principal groups designated Responsive Types, I, II and III respectively.

### *Responsive Type I.*

The responsive Type I tumours repeated the cycle of growth and regression in almost identical form in successive pregnancies. The peaks of growth during pregnancies were at about the same level, and the intervening regressions were usually, but not invariably, complete (Fig. 4, continuous line). The tumour represented by the broken line in Fig. 7 completed 5 cycles with similar peaks and, at the end, was no larger than when it was first seen 18 weeks previously.

FIG. 7.—RB, $F_1$, No. 61. (i) Right groin; (ii) right vulva; (iii) left axilla.

FIG. 8.—BR4 (pink label), $F_4$, No. 211. (i) Left axilla; (ii) left groin; (iii) right vulva.

FIG. 9.—BR4 (pink label), $F_3$, No. 73. (i) Left axilla; (ii) right groin.

The smallest tumour shown in Fig. 8 (broken line) behaved similarly. The peak size, however, was often considerable, as shown in some of the other charts representing Type I tumours (Fig. 20). Some tumours maintained their course unchanged through several months of observation, whereas others after two or three cycles changed their behaviour, as described in a later section.

The growth of Responsive Type I tumours was strictly conditional and dependent on pregnancy. If breeding stopped the tumours disappeared and, as a rule, did not recur until breeding was resumed. The responsiveness to pregnancy and parturition remained constant, and there was no net advance in size or in aggressiveness.

*Responsive Type II.*

The responsive Type II tumours grew to a higher peak at each successive pregnancy but they remained responsive throughout. The range of response at each pregnancy was nearly constant for the same tumour, but varied considerably from one tumour to another. Fig. 7 (continuous line) illustrates a tumour

FIG. 10.—RB, $F_1$, No. 51. (i) Right vulva (? recurrence of tumour excised 7 weeks previously; earlier tumour not shown); (ii) right axilla.

with a small range of growth and regression at each pregnancy, and a slow but steady increase in average size. Another tumour shown in the same diagram (dotted line) appeared 8 weeks after the first and then followed an almost parallel course. The range of pregnancy response and the net growth in each cycle were sometimes much greater, as shown in Fig. 8 and 9. Other examples of Type II tumours are shown in Fig. 1, 2 and 30. Many tumours that were examined only at weekly intervals evidently belonged to this group, although the details of individual cycles were not shown (Fig. 10, continuous line).

The distinctive features of the Type II tumours were their persistent responsiveness and steadily-mounting growth-curves. The peaks of growth, or the averages of the maximum and minimum sizes during successive pregnancies, fell close to a straight line. Thus, the course of a tumour was represented by a curve which could be resolved into a straight line with superimposed waves; the straight line indicated the rate of net growth or, for want of a better term, the *intrinsic growth rate*, and the waves indicated the response to pregnancy and parturition. The intrinsic growth rate as measured by the slope of the straight line, and the responsiveness, as measured by the amplitude of the waves remained constant in Type II tumours; the tumour illustrated in Fig. 7 (continuous line) did not notably change its intrinsic growth rate or its responsiveness during the observation period of 21 weeks. Type II tumours in the same mouse had similar intrinsic growth rates (Fig. 7, 8). The intrinsic growth rate and the degree of response, however, varied within wide limits and independently of each other from one mouse to another. Similar intrinsic growth rates were linked with different degrees of responsiveness and vice versa. Responsive and unresponsive tumours in the same mouse sometimes, as an exception to the general rule, had similar intrinsic growth rates as illustrated in Fig. 10; the wavy curve of the responsive tumour repeatedly crossed the straight line of the unresponsive tumour, but the net growth, after two months, was about the same.

Fig. 11.—BR4 (blue label), $F_4$, No. 179. (i) Right axilla; (ii) right groin.

Fig. 12.—BR6, $F_4$, No. 136. (i) Right neck; (ii) left neck.

*Responsive Type III.*

Responsive Type III tumours maintained a steady average size for considerable periods of time and responded weakly to pregnancy. Their course was represented by a horizontal straight line with superimposed shallow waves (Fig. 11, continuous line). They were rare, in contrast with Types I and II, which were common. Their differentiation from Type I tumours was questionable, but they seemed to correspond with relatively frequent tumours in non-breeding mice whose course was represented by a horizontal straight line, and possibly differed essentially from Type I tumours in the ability to *persist* without the stimulus of pregnancy. The discrepancy in the frequencies in breeding and non-breeding mice might be attributed to the transience of Type III behaviour in breeding mice.

The growth of almost all the tumours encountered in the investigation could be analysed into various sequences of the unresponsive and responsive types already described. The great diversity of behaviour was attributable to two circumstances: first, the intrinsic growth rates and the responsiveness varied within wide limits and independently of each other as shown in the present section and, second, progression from one type to another occurred variously during the clinically manifest course of the tumour. The section which follows deals with progression.

### *Progression.*

### *Progression in responsiveness.*

After courses of varied duration, tumours of responsive Types I and II changed into secondarily unresponsive tumours (Fig. 3, continuous line; Fig. 6, 11, 12, broken lines). The tumours grew according to expectation during a pregnancy but did not regress after parturition; instead, growth continued steadily and progressively. In the diagrams the curve rose to the usual peak

FIG. 13.—BR4 (blue label), $F_4$, No. 24. (i) Left groin; (ii) right groin; (iii) right vulva. Male removed at A, replaced at B.

at the end of pregnancy, and then continued as a straight line somewhat less steep, as a rule, than the ascending limb of the pregnancy wave (Fig. 11, 12). During the last pregnancy shown in Fig. 13 three tumours grew to a peak, and their curves, up to that point, so nearly overlapped that they are distinguished with difficulty in the reduced diagram. Then two tumours declined rapidly from the peak; they were undetected 9 days after parturition, and were not found again during life or post-mortem. The remaining tumour grew progressively from the peak. Evidently the decisive change was in one of three tumours and not in the environment, to which the other two tumours reacted as they had done before.

Those altered tumours that were tested by subsequent pregnancies proved unresponsive (Fig. 12); the other tumours were represented by the curves, approximating to straight lines, characteristic of unresponsive tumours. Growth was often rapid.

With rare and doubtful exceptions, abrupt progression from responsive to unresponsive type was not observed in more than one tumour in the same mouse at the same time.

FIG. 14.—RB, $F_1$, No. 56. (i) Right axilla ; (ii) right groin.

FIG. 15.—BR4 (blue label), $F_4$, No. 37. (ii) Left vulva (earlier tumour (i) not shown ; excised 13 weeks previously ; no recurrence).

FIG. 16.—BR4 (pink label), $F_4$, No. 164. (i) Right groin ; (ii) left axilla.

Rarely, after one or more complete regressions, a responsive tumour recurred as usual during pregnancy and continued its course as a weakly responsive Type III tumour. The change was apparently abrupt, and involved a diminution of the dependence on pregnancy without complete loss of responsiveness (Fig. 14, continuous line). A more gradual loss of responsiveness was sometimes recorded, and resulted in a responsive Type III tumour or a doubtfully unresponsive tumour.

*Progression in intrinsic growth rate.*

The rate of growth of two slow-growing unresponsive tumours changed abruptly (Fig. 15, 16). The acceleration was not attributable to hormonal or other environmental factors for, as shown in Fig. 16, a newly manifested tumour continued the almost horizontal straight line which represented the earlier course of the first tumour. The tumour itself had changed.

The recognition of a similar change in responsive tumours was necessarily more difficult because the pregnancy waves obscured the intrinsic growth rate. Probably an acceleration of growth often accompanied the loss of responsiveness

FIG. 17.—BR4 (pink label), $F_4$, No. 55. (i) Right vulva ; (ii) right vulva (separate from (i) ; (iii) left axilla. (One tumour omitted.) Male removed at A, replaced at B.

described in the preceding section, but the secondarily unresponsive tumours were not necessarily rapid growing. Responsiveness and intrinsic growth rate were independent variables. The difference between the tumours described as responsive Types I and II respectively was more probably in the intrinsic growth rate than in the responsiveness. Some Type I tumours after one or more complete regressions changed into Type II tumours (Fig. 2). The change seemed abrupt and did not abolish the responsiveness. It was manifested at an early or late pregnancy in continuously breeding mice, or at the first or later pregnancy after an interruption of breeding (Fig. 17, 18).

*Interrupted breeding.*

When breeding was interrupted most of the tumours that regressed promptly and completely after the last pregnancy did not recur until breeding was resumed, whereupon, with rare exceptions, they reappeared during the first pregnancy. Some tumours then grew almost exactly as they had done before breeding was stopped ; no progression had occurred in the interim (Fig. 13, 19, 20). Others, however, recurred at the first pregnancy as unresponsive or dubiously responsive tumours (Fig. 21, 25). Many tumours between the extremes recurred with altered

FIG. 18.—BR4 (blue label), $F_4$, No. 47. (i) Left vulva ; (ii) right axilla ; (iii) right groin. (Two tumours omitted.) Male removed at A, replaced at B.

FIG. 19.—BR4 (blue label), $F_3$, No. 12. (i) Left axilla ; (ii) right vulva. (One tumour omitted.) Male removed at A, replaced at B.

FIG. 20.—BR6, $F_4$, No. 49. (i) Left vulva ; (ii) left groin ; (iii) right vulva. Male removed at A, replaced at B.

FIG. 21.—BR4 (pink label), $F_4$, No. 215. (i) Left vulva ; (ii) left groin ; (iii) left vulva (separate from (i)). Male removed at A, replaced at B.

behaviour, a responsive Type I tumour reappearing, for example, as a responsive Type II tumour (Fig. 17). Some tumours, which recurred without evidence of progression, changed at a subsequent pregnancy. The tumour illustrated in Fig. 18 (continuous line) apparently increased its intrinsic growth rate after the second pregnancy following the interruption of breeding.

A minority of tumours recurred during the intermission of breeding, almost immediately or at varied intervals after the last pregnancy. All these recurrent tumours were of unresponsive type (Fig. 22, 23, 24, 26), and they were exactly

FIG. 22.—BR4 (pink label), $F_4$, No. 192. (i) Right axilla ; (ii) left axilla ; (iii) right groin. Male removed at A.

FIG. 23.—BR4 (blue label), $F_4$, No. 144. (i) Right vulva ; (ii) left axilla ; (iii) right groin. Male removed at A. Vasectomized male added at B and replaced by normal male at C.

comparable with the previously-mentioned tumours which recurred in unresponsive form at the first pregnancy when breeding was resumed. When two or three tumours had regressed only one of them recurred as an unresponsive tumour, whether during the intermission of breeding or at the first pregnancy thereafter. Fig. 26 and 25 illustrate tumours which recurred during an intermission and at the succeeding pregnancy respectively. The close resemblance indicated that progression occurred independently of breeding, although the stimulus of pregnancy was often needed to initiate the recurrent growth whereby the change which had already occurred was made manifest.

Tumours that did not regress promptly or completely after the last pregnancy behaved variously during the intermission of breeding. Regression of some

**Fig. 24.**—BR4 (blue label), $F_4$, No. 75. (i) Right vulva; (ii) left vulva. Male removed at A.

**Fig. 25.**—BR4 (blue label), $F_3$, No. 26. (i) Left vulva.

**Fig. 26.**—BR4 (blue label), $F_4$, No. 90. (i) Right vulva; (ii) left axilla. Male removed at A, and replaced at B. Nursed litter from A to B.

**Fig. 27.**—BR4 (pink label), $F_4$, No. 119. (i) Right axilla; (ii) left neck; (iii) left axilla. (One tumour omitted.)

tumours was slow and prolonged but eventually complete (Fig. 17, 19, 22, 23, 28). In continuously breeding mice similar regression would be incomplete when recurrent growth was started by another pregnancy; a consequent summation of the effects of successive pregnancies might account for the ascending waves of responsive Type II tumours. In two tumours shown to the right of Fig. 19, however, the one that regressed completely between pregnancies (broken line) increased its peak size somewhat more steeply than the tumour that regressed incompletely (continuous line). Increasing peak size, therefore, was not attributable, in general, to summation.

The regression of some tumours was slight and of short duration, being followed almost at once by progressive unresponsive growth. The regression was sometimes trivial and probably within the limits of error in measurements, and the tumours were then scarcely distinguishable from those already described which continued as unresponsive tumours from the peak of a pregnancy wave. Possibly the two tumours illustrated in Fig. 27 (broken and dotted lines) that grew progressively after apparent slight regression were primarily unresponsive

FIG. 28.—BR6, $F_4$, No. 74. (i) Right axilla; (ii) left vulva. Male removed at A, replaced at B.

tumours. The regression of other tumours, though brief, was more distinct (Fig. 28, broken line). In yet other tumours the regression was more prolonged, and a gradual decline from the peak size at the last pregnancy merged imperceptibly with an equally gradual resumption of growth, the course being represented by a smooth curve without an indication of abrupt change (Fig. 29, continuous line). The tumour represented by the continuous line in Fig. 29 proved unresponsive at a subsequent pregnancy. Another tumour (broken line) shown in the same figure behaved similarly, except that it apparently began to grow earlier and was less certainly unresponsive at the subsequent pregnancy.

Some tumours regressed slightly or moderately after parturition and then maintained a constant size. The course of these tumours, which possibly corresponded with the responsive Type III tumours in breeding mice, was represented by a horizontal straight line. One of the tumours began to grow again during the intermission of breeding, and continued without evident acceleration through the first pregnancy when breeding was resumed. After a brief trivial regression following parturition, it grew more rapidly during the next pregnancy and was probably unresponsive (Fig. 30, broken line). The remaining tumours persisted without growth until breeding was resumed, and then behaved variously. Fig. 31 (continuous line) and 32 (broken line) illustrate tumours which were

FIG. 29.—BR4 (pink label), $F_4$, No. 21. (i) Left vulva ; (ii) right groin. Male removed at A, replaced at B.

FIG. 30.—BR6, $F_4$, No. 50. (i) Right axilla ; (ii) right vulva ; (iii) left groin. Male removed at A ; cutaneous applications of stilboestrol at B ; vasectomized male added at C and replaced by normal male at D.

FIG. 31.—BR4 (blue label), $F_4$, No. 3. (i) Right groin ; (ii) left axilla ; (iii) left neck. Male removed at A, replaced at B.

probably unresponsive when growth resumed, the irregularities of the growth curves being attributable, most likely, to errors of measurement. The tumour shown in Fig. 33 grew as a responsive Type II tumour with unusually steep ascent. By contrast another tumour (Fig. 34) grew at the first pregnancy to a slight extent within the limits of error of measurement, but grew unresponsively through the second pregnancy and puerperium, and continued to grow unresponsively but more rapidly at the third pregnancy. The terminal course was represented by a straight line, but the intermediate course through the first and second pregnancies after the resumption of breeding was more fairly indicated by a curved line, suggesting a gradual accession of growth energy in an unresponsive (or minimally responsive) tumour.

FIG. 32.—BR4 (blue label), $F_3$, No. 32. (i) Right axilla; (ii) left groin; (iii) right groin. (One tumour omitted.) Male removed at A; injections of theelin at B; male replaced at C.

*Multiple Tumours.*

Most of the mice that did not die prematurely of intercurrent infection or accident developed multiple tumours, up to 7 in number and averaging 2·3 per mouse. Sometimes 2, or less often 3, tumours appeared simultaneously or within a few days of each other (Fig. 5, 6, 22). More commonly the first tumour was solitary and further tumours developed consecutively. So far as could be seen the spacing of successive tumours and the sequence of types were irregular. The ratio of unresponsive to responsive tumours was about the same in first tumours as in second or third tumours. Tumours that remained solitary throughout the period of observation were sometimes unresponsive, but more frequently responsive. In the majority of the mice where two tumours appeared at about the same time both tumours were responsive; in the minority both were unresponsive, or one was responsive and the other unresponsive. As a rule new tumours were registered first during pregnancy.

Different types of tumours developed independently of each other in the same mouse, and progression from one type to another occurred independently in the several tumours. Some mice had, at the same time, 3 tumours of three contrasted types (Fig. 1). Progression occurred without apparent relation to the size or duration of a tumour, and indiscriminately in first, second, or later tumours in

FIG. 33.—BR6, $F_4$, No. 42. (i) Left vulva ; (ii) right vulva. Male removed at A ; cutaneous applications of stilboestrol at B ; vasectomized male added at C and replaced by normal male at D.

FIG. 34.—RB, $F_1$, No. 63. (i) Left groin (earlier tumour omitted). Male removed at A, replaced at B.

FIG. 35.—BR6, $F_4$, No. 11. (i) Left vulva ; (ii) right vulva. Male removed at A ; cutaneous applications of stilboestrol at B ; vasectomized male added at C and replaced by normal male at D.

order of appearance. Abrupt progression from responsive to unresponsive type or from one responsive type to another occurred in only one tumour at a time even when two or more tumours previously had run parallel courses. Exceptions to the rule were rare and equivocal. Development of a new tumour did not noticeably alter the course of tumours already present (Fig. 1, 4). Conversely, the later of consecutive tumours were not evidently regulated by the earlier, for they were as varied in type and as liable to progression as the earlier tumours.

The preceding paragraph stresses independence and irregularity in the behaviour of multiple tumours. Observations of a different kind provide equally impressive examples of parallelism. Many diagrams illustrate parallelism of the pregnancy cycles of responsive tumours (Fig. 7, 8, 9, 13, 17). When the tumours were of similar size the growth curves overlapped so that they were difficult to distinguish in a single diagram (Fig. 13). If the sizes of the tumours and the extents of growth and regression were varied, the peak of growth, nevertheless, was reached simultaneously in all the tumours. Primarily unresponsive tumours grew in parallel (Fig. 5), and so also did primarily and secondarily unresponsive tumours in the same mouse (Fig. 3, 6.) The general rule, with few exceptions, was that *tumours of the same type in the same mouse ran parallel courses.* Tumours of different types, by contrast, did not develop in parallel; the exceptions, though conspicuous, were uncommon, and the parallelism of the intrinsic growth rates of responsive and unresponsive tumours in the same mouse (Fig. 10) was probably coincidental. In view of the extremely wide variability amongst tumours of the same type in different mice, the almost constant parallelism in the same mouse was not plausibly coincidental, but indicated either that multiple tumours were impressed at their origin with similar intrinsic growth rates, or that each was similarly regulated by environmental factors unconnected with reproduction.

### *The Pregnancy Effect.*

### *Growth and regression during pregnancy and the puerperium.*

The consistent feature of the growth curves of responsive tumours was a peak near the time of parturition. The ascent to the peak and the subsequent descent were sometimes remarkably steep (Fig. 23). A tumour illustrated in Fig. 14 completed one cycle of growth and regression within 6 days, and some less distinct tumours were recorded at only one or two of successive daily examinations. The peak of growth was sometimes sharp, the maximum size being maintained for not more than one day, and often rounded or flattened to a plateau lasting two or three days (Fig. 9, 24, 35). Smooth curves were obtained only when the tumours were sharp and regular in outline and conveniently situated. Subsidiary spikes on the less regular curves were probably attributable to inaccurate measurements (Fig. 7).

The most reliable and complete records indicated that the peak of growth was reached during the last day or two of gestation, and that regression began on the day of parturition. A few measurements, made by chance during or immediately after parturition, showed that regression had already started.

Exceptionally, growth seemingly continued for a day or two after parturition (Fig. 26, second pregnancy), but the increments were small, and not certainly outside the limits of error in measurement.

Regression varied widely in speed and extent. Tumours of substantial size ($1 \cdot 5 \times 0 \cdot 9$ cm., Fig. 35) disappeared within 24 hours; others regressed incompletely and more gradually. As indicated by question marks in several figures the persistence, or size, of residual tumours was sometimes doubtful.

Tumours that regressed completely after parturition recurred, as a rule during the second half of the next pregnancy and often during the last week (Fig. 7, 8, broken lines). Sometimes recurrences were not detected until the penultimate or final day of gestation. Tumours that regressed incompletely behaved similarly except that growth was resumed, sometimes, during the first week of pregnancy (Fig. 8, dotted line). When another pregnancy did not follow at once a tumour, shown in Fig. 13, regressed completely within 7 days, but when another pregnancy followed without delay regression was only partial. Possibly hormonal changes during the first 2 or 3 days of pregnancy sufficed to check regression, although growth was not apparent until a few days later.

Perhaps a few of the "recurrent" tumours were, in reality, new primary growths, but so nearly as could be determined the recurrent tumours occupied the same positions as those which had disappeared and were of similar shape. After partial regression the reality of true recurrent growth was not in doubt. Some tumours were reduced to thin plaques not greatly different in superficial dimensions from the tumours at their peaks of growth; recurrence was manifested by a thickening of the whole plaque. Other tumours shrank to vague subcutaneous thickenings of doubtful, but probably considerable, extent; recurrent growth was evidenced by sharpening of the outlines. It seemed that tumours recurred by the simultaneous growth of all parts of a substantial area of previously altered mammary tissue rather than by centrifugal growth from a small residual focus. In consequence, recurrent tumours were often relatively large when first registered and the gradients of growth and regression remarkably steep.

A few tumour-bearing mice nursed and weaned their litters. Their tumours behaved during lactation like those in recently pregnant mice that did not at once become pregnant again although deprived of their young. No specific effect of lactation was seen.

Recurrence was observed, almost always, at each succeeding pregnancy, and most apparent exceptions were attributable to wide spacing of the examinations (Fig. 12). Some dubious tumours, registered once or twice on suspicion, were not seen again, but only 5 tumours, recorded on at least one occasion without query, disappeared permanently from mice that continued to breed. After interruption of breeding the tumours that had regressed recurred, with few exceptions, at the first ensuing pregnancy. Fig. 31 illustrates a tumour (broken line) that recurred doubtfully or not at all.

Some charts showed recurrences of tumours between pregnancies (Fig. 4, 21). The recurrences corresponded as a rule with expected pregnancies in continuously breeding mice, and the recurrent tumours, unlike those in segregated females, were responsive; almost certainly the recurrences were stimulated by unrecorded pregnancies. The general rule was recurrence in each successive pregnancy and no recurrence between pregnancies.

*Mechanism of the pregnancy effect.*

Tumour-bearing females were segregated from males, usually during pregnancy, and, after a period of observation during which tumours regressed partially or completely, they were used to test the effects of pseudo-pregnancy, gonadotropic hormone, and oestrogens. Subsequently most of the mice were mated with normal males to demonstrate the usual pregnancy response.

Eight females were mated with vasectomized males to induce pseudo-pregnancy. Brief recurrence of one tumour was recorded, but it was not comparable in degree with the recurrence at a subsequent normal pregnancy (Fig. 35). With the exception of one doubtful and transitory recurrence, no effect on the tumours was observed in the other mice (Fig. 30, 33).

Pregnancy-urine gonadotropic hormone (Antuitrin S, Parke, Davis & Co.) was injected subcutaneously into 8 mice, three doses each of 0·2 c.c. containing 20 units being given with intervals of 7 and 2 days between. The injections did not induce recurrence of any tumour that had regressed completely; tumours that regressed incompletely responded trivially and doubtfully or not at all.

FIG. 36.—BR4 (pink label), $F_3$, No. 76. (i) Left groin. Male removed at A; injections of theelin at B.

Three experiments were carried out with oestrogens. In the first, each of 8 mice received 4 daily applications to the skin of 0·2 c.c. of a 0·1 per cent solution of diethylstilboestrol in acetone. The solution was old and of doubtful potency. No unequivocal effect was observed (Fig. 33, 35). In the next experiment each of six mice received three subcutaneous injections, at intervals of 7 and 2 days, of 0·05 c.c. of Theelin (Aqueous suspension, Parke, Davis & Co.), and 2 mice received single doses. Each dose contained 1000 units. No tumour that had regressed completely recurred as a result of the injections. The action on tumours that had regressed partially was uncertain. Fig. 32 suggested that the injections possibly delayed regression of one tumour, and Fig. 36 shows regression checked and succeeded by growth at the time of the injections, but as similar events occurred without injections during intermissions of breeding the interpretation was doubtful. In other mice an action on tumours was trivial or lacking.

In the third experiment cholesterol pellets containing 10 per cent of diethylstilboestrol and weighing on the average 8·2 mg. were implanted subcutaneously in 7 mice. Nine tumours previously observed in these mice had disappeared before the pellets were implanted and none recurred promptly, but eventually each mouse developed a solitary tumour. In 3 mice the late tumours recorded

54 days, 145 days and 145 days respectively after implantation of the pellets were in the same region as earlier tumours which had regressed, and were possibly, but, in view of the long delays, not probably, recurrent tumours. In the other 4 mice the late tumours were remote from the sites of the earlier tumours and were undoubtedly new primary growths. They were observed at intervals ranging from 68 days to 216 days after insertion of the pellets. It was doubtful if the oestrogen was responsible for the growth of the late tumours.

None of the experimental procedures reproduced the cycles of growth and regression which occurred in pregnancy and the puerperium. The experiments were indecisive, requiring extension with varied doses and combinations of hormones, but they discounted the possibility that growth during pregnancy was due to an oestrogenic stimulus alone.

FIG. 37.—BR6, $F_4$, No. 90. (i) Right vulva (Tumour A); (ii) left groin (Tumour B).

*Transplantation experiments.*

The transplantation experiments previously recorded (Foulds, 1947, 1949*b*) showed that some tumours grew equally well in male and female hosts, whereas others grew in females or oestrogenized males but not in normal males. No other difference between tumours of the two kinds was observed, but information about the course of the primary tumours was scanty; many tumours were transplanted soon after detection, and the BR $F_1$ tumours together with some of the RB $F_1$ tumours were transplanted before systematic measurement of tumours was begun. The present section records the transplantation of tumours whose previous course was known and describes experiments with tumours removed surgically with three main objectives, namely to study autotransplantation, to compare successive primary tumours in the same host, and to compare primary tumours with the recurrent tumours which followed the operation. The experiments were few in number, the general plan of this investigation being to study the natural course of tumours with a minimum of experimental interference.

Tumours of similar size from the same mouse were used to compare the transplantabilities of responsive and unresponsive tumours. A BR6 $F_4$ mouse (No. 90) had a responsive tumour, A (Fig. 37, continuous line), of 9 weeks' duration; weekly measurements were insufficient to show the true peak of growth in two pregnancies, but the more frequent measurements during the last pregnancy revealed a sharp peak and subsequent regression. Tumour B (broken line) in the same mouse grew during the last pregnancy and without interruption in the puerperium. The tumours were transplanted when almost equal in size. Tumour A grew in female hosts but not in males. In the first passage Tumour B grew equally in male and female hosts, although in the second passage the implants grew tardily in males. Tumours B and C in mouse RC12 (Fig. 38) grew respectively in females only and in males as well as females. Tumour C in mouse RC10

FIG. 38.—RB, $F_1$, No. 84. (i) Right vulva; excised at A, recurred at R (Tumour B); (ii) left axilla (Tumour C). (Two tumours omitted.)

FIG. 39.—RB, $F_1$, No. 36. (i) Right axilla; excised at A; ? recurrence at R (Tumour B); (ii) right groin (Tumour C).

(Fig. 39, broken line) grew in males and females, but Tumour B (continuous line) grew only in females; the difference between males and females was clear-cut, although in the original host Tumour B was only weakly or doubtfully responsive. From these experiments it appeared that, in general, unresponsive tumours were transplantable into male and female hosts and responsive tumours only into female hosts, but that the correlation between type of growth in the primary tumour and behaviour after transplantation was not perfect.

Five tumours were excised and used for autotransplantation, implants being inoculated subcutaneously near the mid-line of the abdomen in order to minimize confusion with new primary tumours. Three of the transplantations were unsuccessful. The primary tumours ranged in size from 0·5 × 0·5 × 0·5 cm. to 1·0 × 0·6 × 0·4 cm., and in duration, from first detection, from 17 to 62 days; each, so far as could be judged from the short period of observation, was "responsive." Two autotransplantations were successful. Growth of the implant from a primary tumour measuring about 0·2 cm. in diameter and removed 6 days after detection was evident after 26 days, and continued steadily until the mouse was killed 22 days later. The primary tumour from another mouse measured 0·6 × 0·4 × 0·2 cm. when excised 24 days after its recognition and was "responsive." The autotransplant was notably responsive during 5 pregnancies, but after the final pregnancy it continued to grow and, apparently, was then unresponsive (Fig. 40, broken line); ulceration of the tumour precluded observation through another pregnancy. This experiment provided evidence of progression in an autotransplant, and suggested that the delayed recurrences and delayed secondary growths observed after the excision of primary cancers from man were attributable to progression in latent tumour deposits.

Transplantation of RB $F_1$ tumours into BR $F_1$ hosts failed 3 times. The tumours ranged in size from 0·8 × 0·7 × 0·5 cm. to 1·2 × 0·9 × 0·5 cm. One of them recurred after operation, grew responsively, and, when transplanted, grew in female but not in male hosts. New primary responsive tumours developed in two of the mice; when transplanted, one of them grew in female hosts and also after pronounced delay in males, and the other grew only in females.

FIG. 40.—RB, $F_1$, No. 62. (i) Left vulva; (ii) right vulva (excised for autotransplantation at E; broken line thereafter represents the autotransplant). Male removed at A, and replaced at B.

Tumours from two RB $F_1$ mice (RB4 and RB10) gave evidence of progression between the transplantation of a primary tumour and subsequent transplantation of a recurrent tumour. Implants of a primary tumour excised from mouse RB4 grew only in females, whereas the recurrent tumour, transplanted 5 weeks later, grew in both males and females. Meanwhile a second primary tumour had developed at a new site. It was excised and transplanted and grew only in females, but a recurrent tumour transplanted 11 weeks later grew in females and also, though more slowly, in males. The primary tumour excised from mouse RB10 (Fig. 39, Tumour A) was transplanted successfully only into female mice, and the resulting tumours were notably influenced by pregnancy (Foulds, 1949*b*). The recurrent tumour (Fig. 39, Tumour B), transplanted 11 weeks later, likewise grew only in females, but the tumours were not responsive to pregnancy.

The experiments showed that in some mice the tumours that appeared first were less adaptable to new hosts than those that developed later, and that some recurrent tumours were more adaptable than their primary growths. The results, however, were not consistent. Successive tumours from 3 RB $F_1$ mice (RB6, RB7 and RB8) grew equally in male as well as female hosts; the primary tumour and two successive recurrences in mouse BR3 alike grew in females and with slight or irregular delays in males; and although the first tumour tested from mouse RB9 grew in both males and females, a later primary tumour grew only in females.

DISCUSSION.

In the light of the present investigation it is not surprising that previous accounts of the behaviour of mammary tumours of mice during pregnancy and the puerperium are conflicting (Slye, 1920; Haddow, 1938; Baatz, 1939). In general, tumours were not followed from their first appearance through successive pregnancies, supposed effects of lactation were not controlled by observations on recently-pregnant mice deprived of their young, and contemporaneous multiple tumours were not compared. Slye combined the measurements of multiple tumours and recorded no details of individual pregnancies. As now shown, different tumours in the same mouse behave variously at the same time and the same tumour behaves variously at different times. Haddow, using in the main dealers' mice of unknown ancestry, recorded frequent measurements during and after pregnancy, and concluded that "no evidence was found to suggest that gestation itself has any influence on the rate of growth of mammary cancer in the mouse, but in approximately half of the available cases it was observed that parturition was followed by a temporary decline in tumour growth rate." Haddow's two figures illustrate, respectively, a substantial though incomplete regression after parturition and a halting of growth without regression; they match some curves of mine. Gardner (1941) described 5 hybrid (C57 black × CBA) $F_1$ mice which, repeatedly, had tumours attaining 1–2 cm. in 2 to 5 pregnancies; the tumours regressed after parturition, but 3 of the mice eventually died with mammary adenocarcinomas.

The responses of the tumours in the hybrid mice used in the present investigation are, possibly, exceptional in frequency and degree, but are not essentially different from those described by Haddow and by Gardner. Notable response to pregnancy and parturition is here recorded in hybrids of varied genetic constitution derived from the inbred strains C57 black, RIII and A; it is not correlated with anomalous transmission of the milk-borne mammary tumour agent.

The pregnancy effect usually becomes apparent during the second half of gestation, but probably begins during the first week and continues until the day before parturition. The abrupt regressions about the time of parturition suggest the sudden withdrawal of a stimulus which, though not yet identified, is presumably hormonal, affecting widely-distributed multiple tumours. The preliminary experiments with hormones are indecisive; a simple oestrogenic action seems unlikely, and the possible effect of gonadotropic hormones, placental hormones, and the co-operative action of oestrogenic and luteal hormones require further investigation. Lactation has no specific effect, and modifies the course of the tumours only in so far as it delays the next pregnancy.

The pregnancy effect on mammary tumours is comparable with the "promoting" action of various procedures on chemically-induced tumours of the skin of rabbits and mice. Rous and his colleagues (Rous and Kidd, 1941; MacKenzie and Rous, 1941; Friedewald and Rous, 1944*a*, *b*) studied promoting factors in rabbits, and Berenblum, who has recently adopted Rous's nomenclature, in mice (Berenblum, 1944; Berenblum and Shubik, 1947). Rous and his colleagues describe the "promoting" action of trauma and irritants in eliciting and maintaining tumour-growth from latent tumour cells present in the skin as a result of the "initiating" action of a carcinogen. Some "conditional" tumours grow only whilst the promoting factor operates; they disappear when it is withdrawn, but recur from the irreversibly altered epithelum when the promoting factor is restored. Rous and Kidd discuss whether or not the conditional growths of rabbits' skin are "true tumours." Their argument applies equally to the conditional mammary tumours of mice. In essential agreement with Rous and Kidd, I consider that their exclusion from the group of tumours by an arbitrary definition is unwarranted, and I describe as "tumours" all the mammary growths except a few palpable nodules which regress and do not recur, and those nodules, probably similar, that do not survive autotransplantation. They are accountable to a reversible change in mammary tissue, and perhaps to the exceptional persistence in gross nodules of the properties of the hyperplastic nodules or "adenomas" which are widely accepted as precursors of mammary tumours in mice although many of them are abortive (Gardner, 1941). Browning (1948) reported that nodules less than 0·2 cm. in diameter in $C_3H$ mice were not transplantable, and that about a third of them regressed, whereas nodules larger than 0·2 cm. in diameter never regressed. My observations differ from Browning's in revealing no correlation between behaviour and size; autotransplantation of tumours more than 0·5 cm. in diameter was unsuccessful.

The remaining mammary tumours develop from an irreversibly altered mammary tissue. The great diversity of behaviour is attributable to the interplay of factors which vary within wide limits and independently of each other. Classification into types, though convenient for description, is arbitrary, and hampered by gradations from one type to another. The primary distinction, although not absolute, is between the "responsive" tumours whose course is notably influenced by pregnancy and parturition and the "unresponsive" tumours whose growth is unaffected by reproduction.

The tumours assigned to Responsive Type I correspond with the "conditional" neoplasms of rabbits' skin. They grow during pregnancy, regress promptly after parturition, and recur, with rare exceptions, in the same place at each successive pregnancy; after several cycles of growth and regression they achieve no net increase in size or aggressiveness. The Responsive Type II tumours are less strictly "conditional"; they wax and wane at each gestation, but with a net increase of size at each cycle. The rate of net increase is steady and characteristic of the individual tumour, depending on a specific property described as the "intrinsic growth rate." The term is provisional, to be discarded when elucidation of the property suggests a better name, and is used to denote the steady growth, represented by a straight line, upon which the waves of response to pregnancy are superimposed. The intrinsic growth rate, measured by the slope of the straight line and the responsiveness as indicated by the amplitude of the pregnancy waves, are constant, often for long periods, in a particular tumour, but vary within wide limits and independently of each other from one tumour to another. The Responsive Type III tumours, of more doubtful significance, suggest a capacity for *persistence* but not for growth in the absence of promoting stimuli.

Unresponsive tumours grow progressively without acceleration during pregnancy or retardation in the puerperium. Often they grow rapidly, but growth may be extremely slow. Responsiveness to pregnancy and intrinsic growth rate are independent variables.

Multiple tumours are of the same or of different responsive or unresponsive types, with similar or diverse intrinsic growth rates. There is no apparent regularity in the distribution of types amongst simultaneous or consecutive multiple tumours. They behave, as it seems, in complete independence of each other except that tumours of the same type in the same mouse usually follow parallel courses. This applies to unresponsive and responsive tumours alike. Tumours of the same type in the same mouse either have similar intrinsic growth rates, impressed at their origin, or they are subject to an unidentified regulation by their environment, unconnected with reproduction. " Unresponsive " is used, in this paper, in a special sense to describe tumours which do not respond to the physiological changes of pregnancy and the puerperium; it does not preclude response to other physiological or pathological stimuli.

Independent variation in responsiveness and in intrinsic growth rate accounts, in large measure, for the observed diversity of behaviour, the remaining factor of importance being progression.

I define progression as *irreversible qualitative change* in tumours. It is distinct from progressive growth or extension without qualitative changes, and from reversible alterations in structure or behaviour occasioned by extraneous stimuli. Progression is probably of frequent occurrence and fundamental importance in tumours of animals and of man, but hitherto it has not been specifically defined or analysed. The most explicit references to progressive qualitative changes in tumours are provided by Rous and Kidd (1941) and by Berenblum (1944) in their descriptions of step-like development of induced skin tumours of rabbits and mice, by Greene (1940) in his account of " progressive steps in a graded evolutionary progress " of mammary tumours in rabbits, and in a recent summary of observations on uterine tumours of rabbits (Greene and Newton, 1948) and by Browning (1948) in a study of mammary tumours in C3H mice. Greene and his colleagues and Browning detect progression mainly by transplantation experiments, and in particular by heterotransplantation into the anterior chamber of the eye, and they use the capacity for heterologous growth in the anterior chamber of the eye as a measure of independence or " autonomy " of tumours. The behaviour of a tumour in its natural host is conditioned by its independence of factors operating in that host. I am not satisfied that heterotransplantation measures the same kind of independence. Experiments with mouse tumours do not establish a consistent relationship between clinical behaviour and the results of heterotransplantation (Dyer and Kelly, 1946). Although transplantation experiments disclose progressive changes in tumours, they are not substitutes for observation of the natural course of a tumour in its original host.

A detailed review of these and other numerous and diverse indications of qualitative changes during the growth of tumours is beyond the scope of this paper; it must suffice to mention, as additional examples, the frequent qualitative changes in transplanted tumours, and, notably, in transplanted mammary fibro-adenomata in rats, in oestrogen-induced tumours of the testis in mice (Hooker and Pfeiffer, 1942), and, as I shall describe elsewhere, in vesical tumours induced in mice by 2-acetylaminofluorene. It is here proposed that these diverse phenomena, and many others, are manifestations of a fundamental characteristic of tumours, namely the capacity for progression, as above defined, and that general principles or rules of progression, deduced from the study of mammary tumours of mice, are widely applicable.

The material for this analysis of progression consists of spontaneous tumours following their natural courses without experimental interference beyond temporary interruption of breeding. It is especially favourable on account of the abundance of multiple tumours, for the comparison of multiple tumours establishes beyond doubt that progression is a change in the individual tumour ; alteration in only one of several contemporaneous growths is not attributable to change in a hypothetical " general resistance."

The varied tumours encountered in this investigation may be arranged in a series having at one extreme the wholly conditional responsive tumours, at the other extreme the rapid-growing unresponsive tumours, and intermediately all degrees and combinations of responsiveness and intrinsic growth rate. Individual tumours, however, do not in general traverse this series ; they advance by abrupt steps, vaulting many possible transitional stages. At its first clinical manifestation a tumour may occupy any position in the series of types, irrespective of the time of its occurence or its position in a sequence of multiple tumours. Tumours which develop simultaneously or consecutively in the same mouse conform sometimes to the same type and sometimes to as many types as there are tumours. Progression occurs independently and unpredictably in multiple tumours in the same animal irrespective of their size or clinical duration. Progression may occur earliest in the first, second, or any subsequent member of a sequence of multiple tumours. When two or three tumours, similar in size, duration, and type of behaviour are present, progression occurs in only one of them at a time. Moreover, the progression of one tumour has no apparent effect on the course of the others. The present observations decisively controvert Browning's (1948) suggestion that a second tumour may " abrogate the autonomy " of the first.

The several recognizable characters of individual tumours undergo progression independently. Often, no doubt, responsiveness and intrinsic growth rate change simultaneously, but in some tumours progression in responsiveness occurs without progression in intrinsic growth rate and vice versa. Transplantation experiments provide further evidence of independent progression of characters, namely the responsiveness of transplanted tumours to pregnancy and the gross milky secretion in response to oestrogenic stimulation (Foulds, 1949*b*).

The development of a tumour does not always reach its end-point within the life-time of the host ; progression advances further during serial transplantation (Foulds, 1949*b*). Though difficult to prove, progression from responsive to unresponsive type is probably inevitable if serial transplantation is sufficiently prolonged. Transplantation, in consequence, is of limited value for determining the stage of progression of a primary tumour, since the experimental procedure may occasion further progression.

The recurrence of previously responsive tumours as unresponsive ones indicates that progression advances in responsive tumours whose growth is suppressed. Progression is independent of growth. Moreover, pregnancy is not the sole or essential cause of progression, which is manifested sometimes during intermissions of breeding and, generally, in only one of multiple tumours exposed to the same pregnancies. Pregnancy, however, usually discloses, and possibly accelerates progression ; it seems to exert a "trigger-action." The observations in general favour the hypothesis that the hormonal conditions of pregnancy and the puerperium determine whether or not a tumour shall grow ; they do not decide *how* it shall grow. The course of the growth elicited by pregnancy depends on specific properties which change, by progression, in latent tumour cells and in stationary tumours.

The manifestation of progression in the mammary tumours is usually abrupt, as it is in the induced skin tumours of rabbits and mice, but progression itself is not necessarily abrupt ; it is possible that cumulative gradual changes reach a threshold level at which they are first manifest. Some tumours evidence a gradual progression through a graded sequence of types. Gradual, continuous progression seems uncommon, but it is less conspicuous than the abrupt type, and more accurate and more frequent measurements might reveal it more frequently. Although the observations are few and indecisive, it seems that slow gradual progression is apt to result in sluggish, irregular, or dubiously unresponsive growth. The unequivocal rapid-growing unresponsive tumours, as a rule, are either primarily unresponsive or the result of abrupt progression from a fully responsive and, often, strictly conditional tumour. Apparently two paths of progression are available. One path leads directly to unresponsive tumour ; the tumour acquires its definitive properties early without traversing the numerous intermediate stages which are possible, and which are observed on the other path or "responsive detour." The detour leads ultimately to an unresponsive tumour, but progression along it may be slow and gradual and the end-point indeterminate, or reached only after transplantation. Abrupt progression switches many tumours from the detour to the direct path ; jumping the intermediate stages, the tumours change from the fully responsive to the definitive unresponsive type. The change of path seems the less frequent and the less decisive the further the tumour has travelled along the responsive detour. Under some circumstances at least, as shown in the experiment with stilboestrol pellets in non-breeding mice, neoplastic mammary tissue which has entered the responsive detour is less liable to develop into a progressive unresponsive tumour than mammary tissue that, at the corresponding time, is unaltered or has not progressed beyond the subthreshhold neoplastic state. It is concluded that progression may be continuous, by gradual change, or discontinuous by abrupt and often long steps, and that alternative paths of development are open.

To summarize, I propose the following Rules of Progression :

*Rule I.—Independent progression of multiple tumours.* Progression occurs independently in different tumours in the same animal.

*Rule II.—Independent progression of characters.* Progression occurs independently in different characters in the same tumour.

*Rule III.—Progression is independent of growth.* Progression occurs in latent tumour cells and in tumours whose growth is arrested. Two notable corollaries of Rule III are :

(i) At its first clinical manifestation a tumour may be at any stage of progression.

(ii) Progression is independent of the size or clinical duration of a tumour.

*Rule IV.*—Progression is *continuous or discontinuous*, by gradual change or by abrupt steps.

*Rule V.*—Progression follows one of *alternative paths of development.*

*Rule VI.*—Progression does not always reach an end-point within the lifetime of the host.

These principles, as I believe, are widely applicable to the behaviour of tumours in animals and in man. In particular they account for many vagaries which have encouraged the proposition of speculative alternatives to the orthodox pathology of tumours such as that of Delarue (1947). A few applications are here briefly indicated.

The behaviour of tumours is the resultant of multiple characters which vary, within wide limits, independently of each other and undergo independent progression. The characters include growth energy, invasiveness, capacity for dissemination, and responsiveness to environmental stimuli, of which the hormones are the most easily recognized, but not necessarily the only, examples. " Malignancy " is not a single character. The typical malignant tumour of the text-books is the result of proportionate development of all the characters proper to malignant tumours. Independent progression of characters (Rule II), however, results in disproportionate or " out-of-step " development, as for example in the " benign " tumours that metastasize and the " locally malignant " tumours that do not. The out-of-step development is especially important in carcinoma of the prostate, which, despite conspicuous growth, invasion and dissemination, is responsive to hormones. Willis (1948) criticizes the sharp division of tumours into innocent and malignant groups, and deprecates the question, " Is this tumour innocent or malignant? " ; the appropriate question in his opinion is, " How innocent or malignant is this tumour ? " It is even more important, I suggest, to ask, " In which characters, and to what degree in each of them, is the tumour innocent or malignant? "

Errors in the prognosis of " early " tumours are explained by the observations that progression is independent of the size or duration of a tumour, and that progression occurs without manifest growth, as in latent or stationary tumours (Rule III and corollaries). A tumour may be small in size and young in clinical duration, yet far advanced in the progression of aggressive characters. Progression without manifest growth and independently of the size or duration of tumours probably accounts, too, for the otherwise mysterious but clinically important phenomena of long-delayed recurrence and secondary growth after apparent " cure " of primary tumours. Similarly the ultimate failure of several chemotherapeutic methods after favourable early response is reasonably attributed to progression in the inhibited tumours. The analogy with the recurrence of a previously responsive mammary tumour as an unresponsive growth, during intermissions of breeding in mice, is impressive. Further elucidation of progression, especially the independent progression of characters and progression when growth is inhibited, is evidently of prime importance for the management of human cancer.

### SUMMARY.

Spontaneous mammary tumours in 275 hybrid mice were measured repeatedly from their first appearance until the death of the mice. Two main types of behaviour were distinguished : " *unresponsive tumours* " grew steadily without deviation during pregnancy or the puerperium, whereas " *responsive tumours* " grew during pregnancy to a peak shortly before parturition, thereafter regressed, and recurred at the next pregnancy. Some responsive tumours (Type I) attained similar peaks in successive pregnancies, but others (Type II) reached successively higher peaks, the rate of net growth or " *intrinsic growth rate* " remaining constant. Responsiveness and intrinsic growth rate were independent variables, and were variously combined in individual tumours. Some tumours maintained the same type of behaviour throughout their clinical course ; others changed their course, often abruptly, as the result of an irreversible qualitative change in the tumours termed " *progression*." The diversity of behaviour of the spontaneous tumours was attributable, in the main, to varied combinations of responsiveness and intrinsic growth rate, and to progression in one or both of these characters. The following generalizations or *rules of progression* summarize the results of the analysis of the behaviour of tumours, especially multiple tumours, in breeding and non-breeding mice: (1) Progression occurs independently in different tumours in the same animal. (Independent progression of multiple tumours.) (2) Progression occurs independently in different characters in the same tumour. (Independent progression of characters.) (3) Progression is independent of growth, occurring in latent tumour cells and in stationary tumours. (4) Progression is continuous or discontinuous. (5) Progression follows one of alternative paths. (6) Progression of a tumour does not always reach its end-point within the lifetime of the animal.

These rules are probably widely applicable to the behaviour of varied tumours in animals and in man, and some applications are mentioned briefly. " Malignancy " is not a single character. Disproportionate or " out-of-step " development of the various characters which determine malignant behaviour (independent progression of characters) accounts for many anomalies in the behaviour of cancer in man, and progression in tumours whose growth is inhibited probably explains the ultimate failure of therapeutic measures which have a favourable immediate effect.

I am grateful to Mr. L. E. G. Miall for assiduous technical assistance.

### REFERENCES.

Baatz, H.—(1939) *Geburtsh. Gynäk.*, **118**, 124.
Berenblum, I.—(1944) *Arch. Path.*, **38**, 233.
*Idem* and Shubik, P.—(1947) *Brit. J. Cancer*, **1**, 379.
Browning, H. C.—(1948) *J. nat. Cancer Inst.*, **8**, 173.
Delarue, J.—(1947) ' La problème biologique du Cancer.' Paris (Masson et Cie).
Dyer, H. M., and Kelly, M. G.—(1946).—*J. nat. Cancer Inst.*, **7**, 177.
Foulds, L.—(1947) *Brit. J. Cancer*, **1**, 362.—(1949*a*) *Ibid.* **3**, 230.—(1949*b*) *Ibid.* **3**, 240.
Friedewald, W. F., and Rous, P.—(1944*a*) *J. exp. Med.*, **80**, 101.—(1944*b*) *Ibid.*, **80**, 127.
Gardner, W. U.—1941) *Cancer Res.*, **1**, 345.
Greene, H. S. N.—(1940) *J. exp. Med.*, **71**, 305.
*Idem* and Newton, B. L.—(1948) *Cancer*, **1**, 82.
Haddow, A.—(1938) *J. Path. Bact.*, **47**, 553.
Hooker, C. W., and Pfeiffer, C. A.—(1942) *Cancer Res.*, **2**, 759.
MacKenzie, I., and Rous, P.—(1941) *J. exp. Med.*, **73**, 391.
Rous, P., and Kidd, J. G.—(1941) *Ibid.*, **73**, 365.
Slye, M.—(1920) *J. Cancer Res.*, **5**, 25.
Willis, R. A.—(1948) 'Pathology of Tumours.' London (Butterworth & Co.).